# On the sensitivity of plankton ecosystem models to the formulation of zooplankton grazing

**DOI:** 10.1371/journal.pone.0252033

**Published:** 2021-05-25

**Authors:** Fanny Chenillat, Pascal Rivière, Mark D. Ohman

**Affiliations:** 1 Laboratoire des Sciences de l’Environnement Marin (LEMAR), Université de Brest, Ifremer, IRD, IUEM, Brest, France; 2 Scripps Institution of Oceanography, University of California, San Diego, La Jolla, California, United States of America; Stockholm University, SWEDEN

## Abstract

Model representations of plankton structure and dynamics have consequences for a broad spectrum of ocean processes. Here we focus on the representation of zooplankton and their grazing dynamics in such models. It remains unclear whether phytoplankton community composition, growth rates, and spatial patterns in plankton ecosystem models are especially sensitive to the specific means of representing zooplankton grazing. We conduct a series of numerical experiments that explicitly address this question. We focus our study on the form of the functional response to changes in prey density, including the formulation of a grazing refuge. We use a contemporary biogeochemical model based on continuum size-structured organization, including phytoplankton diversity, coupled to a physical model of the California Current System. This region is of particular interest because it exhibits strong spatial gradients. We find that small changes in grazing refuge formulation across a range of plausible functional forms drive fundamental differences in spatial patterns of plankton concentrations, species richness, pathways of grazing fluxes, and underlying seasonal cycles. An explicit grazing refuge, with refuge prey concentration dependent on grazers’ body size, using allometric scaling, is likely to provide more coherent plankton ecosystem dynamics compared to classic formulations or size-independent threshold refugia. We recommend that future plankton ecosystem models pay particular attention to the grazing formulation and implement a threshold refuge incorporating size-dependence, and we call for a new suite of experimental grazing studies.

## Introduction

Marine plankton dynamics are key determinants of ocean processes ranging from biogeochemical cycling [1], sustainable fisheries [2], and persistence of endangered and protected species, to local [3] and global [4,5] patterns of ocean biodiversity. Phytoplankton growth can alter ocean heat budgets [6]), and zooplankton behavior can potentially modify turbulent mixing [7]. Hence, model representations of plankton structure and dynamics have consequences for a broad spectrum of contemporary issues. Yet, representing the structure of plankton ecosystems in tractable ocean models can be daunting, since holoplanktonic organisms include several thousand species [8,9], untold numbers of Operational Taxonomic Units, at least six trophic levels, and widespread mixotrophy [10]. Moreover, such organisms span a size range (as biovolume) of at least 16 orders of magnitude, a particularly relevant point because predator-prey interactions are thought to be at least partially size-dependent [11]. Approaches taken to simplify representation of this taxonomic, trophic, and size diversity range from early NPZ (nutrient-phytoplankton-zooplankton) models [12–14] that compressed all dynamics to three internally uniform trophic levels, to plankton functional types [15–19] and trait-based approaches (Martini et al. 2020 [20] and references within). Some have represented phytoplankton size classes with allometrically scaled relationships [21–25], while ‘self-organizing’ models permit emergence of phytoplankton types of different physiological characteristics [4,26]. Among these approaches, allometric scaling has been appealing to many because the number of free parameters in a model can be dramatically reduced.

Since many important scientific issues require analysis on large spatial scales, plankton ecosystem models are increasingly embedded in Global Circulation Models. This scale is necessary to address, for example, ocean carbon sequestration partly mediated by the planktonic food web in the Biological Carbon Pump and sustainable fisheries production. However, to date such global scale models have been more successful at reproducing primary production and spatial distributions of plankton types in the open ocean [4,24,25,27] than in the dynamic boundary currents at the eastern and western margins of the ocean basins. Eastern Boundary Currents, in particular, are regions of elevated and highly dynamic carbon fluxes [28,29] and fisheries production [30], suggesting that plankton growth and composition in such regions need to be much better represented than currently found in global models.

Although it is common to explain the deficiencies of biogeochemical model fits by pointing to the more complex physics and need for higher spatial resolution in Eastern Boundary Currents [29,31], here we draw attention, instead, to the critical importance of more accurate representation of biological processes. We focus, in particular, on the zooplankton and their grazing dynamics. Historically, much of the development of plankton ecosystem modeling has emphasized phytoplankton growth, with the assumption that phytoplankton temporal dynamics and spatial patterns are governed primarily by responses to temperature, light, and nutrients (including trace metals). The effects of these variables on phytoplankton growth and species composition are generally well constrained by laboratory experiments and readily incorporated into model structures (e.g., [4,18,24,32]), although interaction terms among variables need further attention (e.g., [33]). In contrast to controls on the specific growth rate of phytoplankton, loss terms, principally grazing by zooplankton [34] but also viral lysis and sinking, are typically represented in a highly simplified form. In many respects, this imbalance is surprising, since the functional response of zooplankton grazing to changes in prey density is known to markedly affect phytoplankton dynamics (e.g.,[12,27,35–40]) and top-down grazing control has been suggested to play a fundamental role in productive ecosystems [24]. In addition, the choice of mathematical formulation of grazing, including subtle differences, has been shown to induce large deviations in the plankton model response [12,27,41,42]. Nevertheless, the imbalance of effort and attention to growth processes over grazing losses persists in the literature, especially for models applied on the large scale (cf. [4]).

A consequence of this imbalanced effort is that it remains unclear whether the specific means of representing zooplankton grazing is likely to have a governing role for phytoplankton community composition, growth rates, and spatial patterns in contemporary, spatially-resolved plankton ecosystem models. Here we conduct a series of numerical experiments that explicitly address this question in order to assess the sensitivity of phytoplankton spatial distributions, community composition, and food web energy flow to the formulation of zooplankton grazing. We do not seek to explore detailed taxon-specific grazing behaviors (e.g., [43]), which are currently computationally intractable for global-scale models, nor do we attempt to expand the number and diversity of types of modeled zooplankton represented. Instead, we focus on a universal aspect of phytoplankton-grazer interactions, i.e., the form of the functional response to changes in prey density, including the formulation of a grazing refuge [41,42]. We evaluate the consequences that alternative representations may have for key ecosystem processes. Anderson et al. [27] analyzed the consequences of four different functional responses for plankton spatial distributions, primary production, and carbon export, using two zooplankton groups, but did not explicitly test sensitivity to grazing refugia.

We conduct a process-oriented numerical study with a size-structured plankton ecosystem model that has been previously calibrated and validated on a global scale [24]. We perform the numerical simulations with a physical model of the California Current System (CCS), an Eastern Boundary Current upwelling ecosystem where better representation of food web processes is needed. In the context of this process study, we analyze the response of the ecosystem in terms of structure and functioning by varying only the form of the grazing term. We are thus able to quantify the changes induced by such process and we show that an explicit grazing refuge, with refuge prey concentration dependent on grazers’ body size, is likely to provide more coherent plankton ecosystem dynamics compared to more classical formulations as in the original model.

## Materials and methods

In this study we adopt a process study methodology to focus on the effects of grazing formulation on ecosystem structure and functioning. We use a physical regional model that was previously validated in the California Current System to force a continuum size-structured ecosystem model. The ecosystem model was previously calibrated and validated on global scale. Our goal is not to reproduce the real ecosystem dynamics by calibrating or finely tuning the model to specific field conditions, but rather to study its sensitivity to the grazing formulation. This is why we only change the grazing term of the biological model equations; all other terms and parameters being kept to their original forms and values. We describe and quantify the consequences of different grazing formulations on the ecosystem dynamics in terms of Chl-*a* distribution, seasonal plankton biomass evolution, trophic interactions and plankton spatial niches and richness.

### Model configuration

#### Physical model

We use the Regional Ocean Modeling System (ROMS), a hydrodynamic model that is a three dimensional, free-surface, hydrostatic, eddy-resolving primitive equation ocean model based on the Boussinesq approximation and hydrostatic vertical momentum balance [44]. The model configuration is the same that Chenillat et al. [45] successfully used with an NPZD-type model over the entire CCS, from the coast to 1000 km offshore and from Baja California (24°N) to Vancouver Island (50°N). It has a 15 km horizontal resolution with 32 sigma-coordinate levels irregularly spaced with higher resolution near the surface to adequately resolve upper ocean physics and ecosystem dynamics. We use monthly mean climatologies to force the model at the surface and its lateral boundaries, as in Chenillat et al. [46,47].

After a 10-year physical spin-up, the coupled biological-physical model is integrated forward for 11 years. Initial and boundary conditions for plankton biomasses and nitrogen-based nutrients are taken from Chenillat et al. [45]; total phytoplankton biomass and total zooplankton biomass were distributed equally among initialized compartments. The ecosystem model gets adjusted after 5 years (not shown). All the results herein are based upon the last 6 years of integration.

#### Continuum size-structured plankton ecosystem model

**Fig 1:**
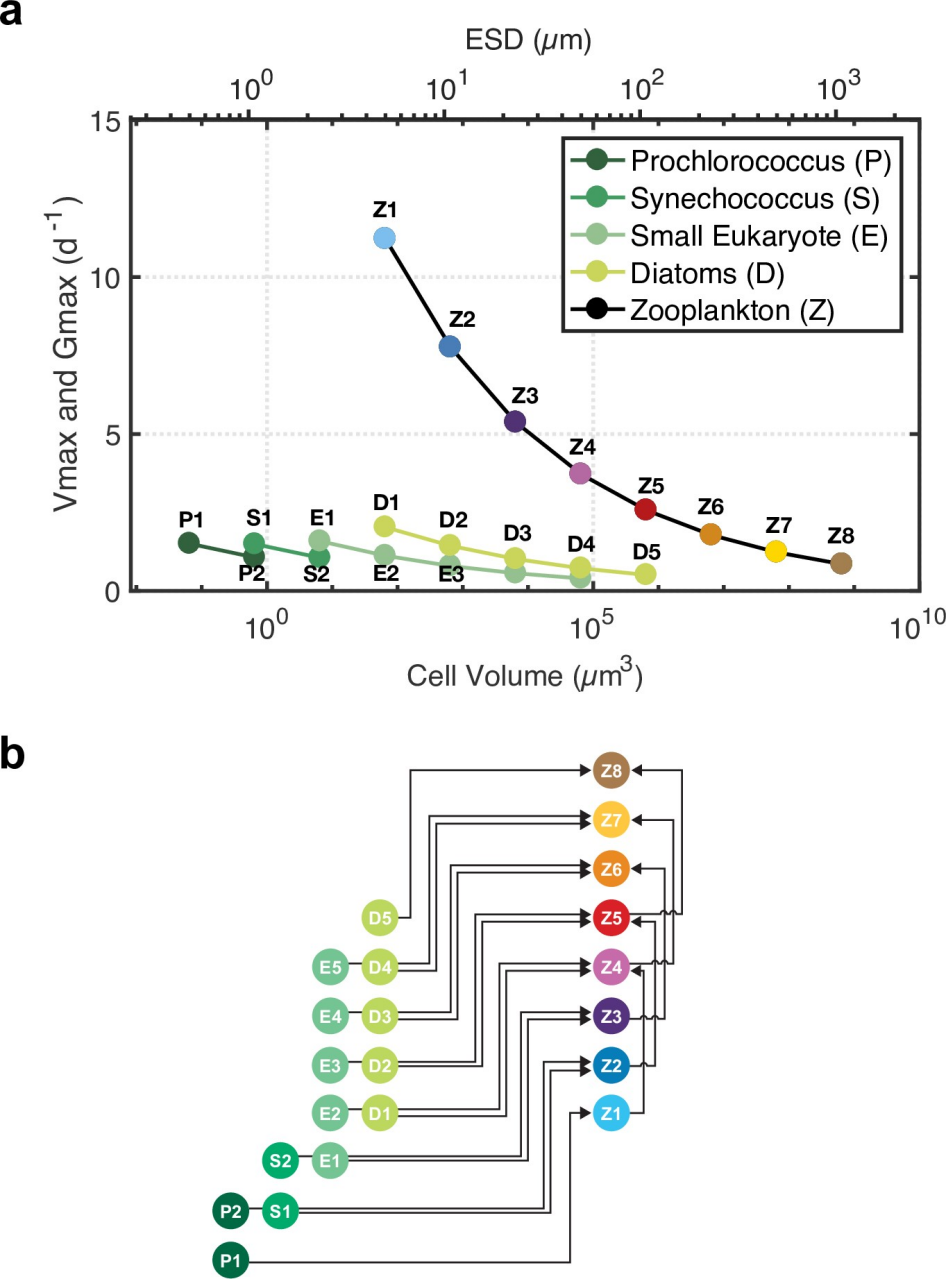
(a) Maximum phytoplankton growth rates and maximum zooplankton grazing rates as a function of cell size (volume and equivalent spherical diameter, ESD) and taxon. (b) Schematic representation of the ecosystem model simplified to show only grazing and predation fluxes for clarity (for a complete representation of the model, see Fig 2 in Ward et al. (2012) [24]). Phytoplankton functional types (*Prochlorococcus* (P), *Synechococcus* (S), small Eukaryotes (E), Diatoms (D)) are divided into different size classes (P1, P2, S1, S2, etc.). Phytoplankton and zooplankton size classes (Z1, Z2, Z3, etc.) are represented with a color pallet used in subsequent figures.

We use the ecosystem model of Ward et al. [24] that describes the temporal dynamics of four functional groups of phytoplankton and one zooplankton group, all further resolved into a continuum of size classes. The model and equations are specified in Ward et al. [24] and its appendix. The four phytoplankton functional types (pft) are analogs of *Prochlorococcus*, *Synechococcus*, small eukaryotes (non-diatoms), and diatoms; each pft is divided into 2 (called P1 and P2), 2 (S1 and S2), 5 (E1 to E5) and 5 (D1 to D5) size classes, respectively. Thus, 14 size classes of phytoplankton ranging from 0.5 to ~100 μm equivalent spherical diameter (ESD, (Fig 1A)) are initialized. The zooplankton are divided into eight size classes (Z1 to Z8, ranging from ~5 to 1000 μm ESD) (Fig 1A): Z1 to Z3 are nanozooplankton-like, Z4 and Z5 microzooplankton-like, and Z6 to Z8 mesozooplankton-like. Each size class is twice the volume of the previous size class. This model is based on carbon, nitrogen and iron. For their growth, phytoplankton access multiple inorganic nutrients, including nitrate (except *Prochlorococcus*), nitrite, ammonium, and iron. Model equations and parameters are taken from the Ward et al. [24] configuration that has been applied globally. We use this familiar formulation despite the many simplifications inherent to such a model structure (*cf*. [43]), so as to focus in detail on the structure and consequences of the grazing formulation. One distinctive feature of this model is that phytoplankton growth is a light- and temperature-dependent function of intracellular nutrient reserves, i.e., of cell quotas [48,49]. Because such a quota formulation is known to better represent plankton dynamics and nutrient cycling [50,51], the modelling community is showing growing interest in quota formulation in biogeochemical models at the global scale (e.g., with PISCES-QUOTA [18]). For clarity, below, we call the model QUOTA.

Available inorganic nutrients are used for phytoplankton growth, and phytoplankton are grazed by zooplankton. Processes such as mortality, sloppy feeding, and egestion allow the transfer of living organic material into sinking particulate detritus and dissolved organic matter. Organic detritus is returned to inorganic form through a simple parameterization of bacterial remineralization.

Following previous work that uses continuum size-structured plankton ecosystem models [21,22], the QUOTA model uses allometric relationships. Allometric relationships use a power-law function that links physiological traits *p* to cell volume *V*, thus reducing the number of free parameters in such a complex model, as follows:

$$p=aV^{b}$$ (1)

*a* and *b* are the allometric parameters, with *b* describing the size dependency. In QUOTA, allometric relationships are used for physiological traits such as nutrient uptake, quota size, growth, mortality, sinking, and grazing rates. For most of these processes, *a* is held constant among phytoplankton groups except for maximum growth rates which vary with taxonomic group [32,52]. Within each pft, larger cells have a slower growth rate due to the allometric relationships, but among cells of similar size, diatoms will grow faster than, for example, small eukaryotes (Fig 1A). As in the original version of QUOTA, the maximum grazing rate follows allometric relationships defined by Hansen et al. [53] as a function of cell volume and we follow the Ward et al. [24] parameter choices (Fig 1A), although we find the potential maximum rates to be rather elevated in some cases. Zooplankton can feed on either phytoplankton or zooplankton, or some combination of both. Zooplankton attack rate is not modified by the density of individual prey types [39]; however, zooplankton feed preferentially on the denser prey between phytoplankton and zooplankton. Each zooplankton size class has a prey size preference because smaller prey are less likely to escape and are easier to ingest. A size-specific predator can thus feed on a variety of prey (zooplankton or phytoplankton from different pft, Fig 1B). This preference is set to a log-normal distribution centered to a predator to prey length scale of 10 and a standard deviation of 0.5 [11].

#### Grazing refuge formulation

**Fig 2:**
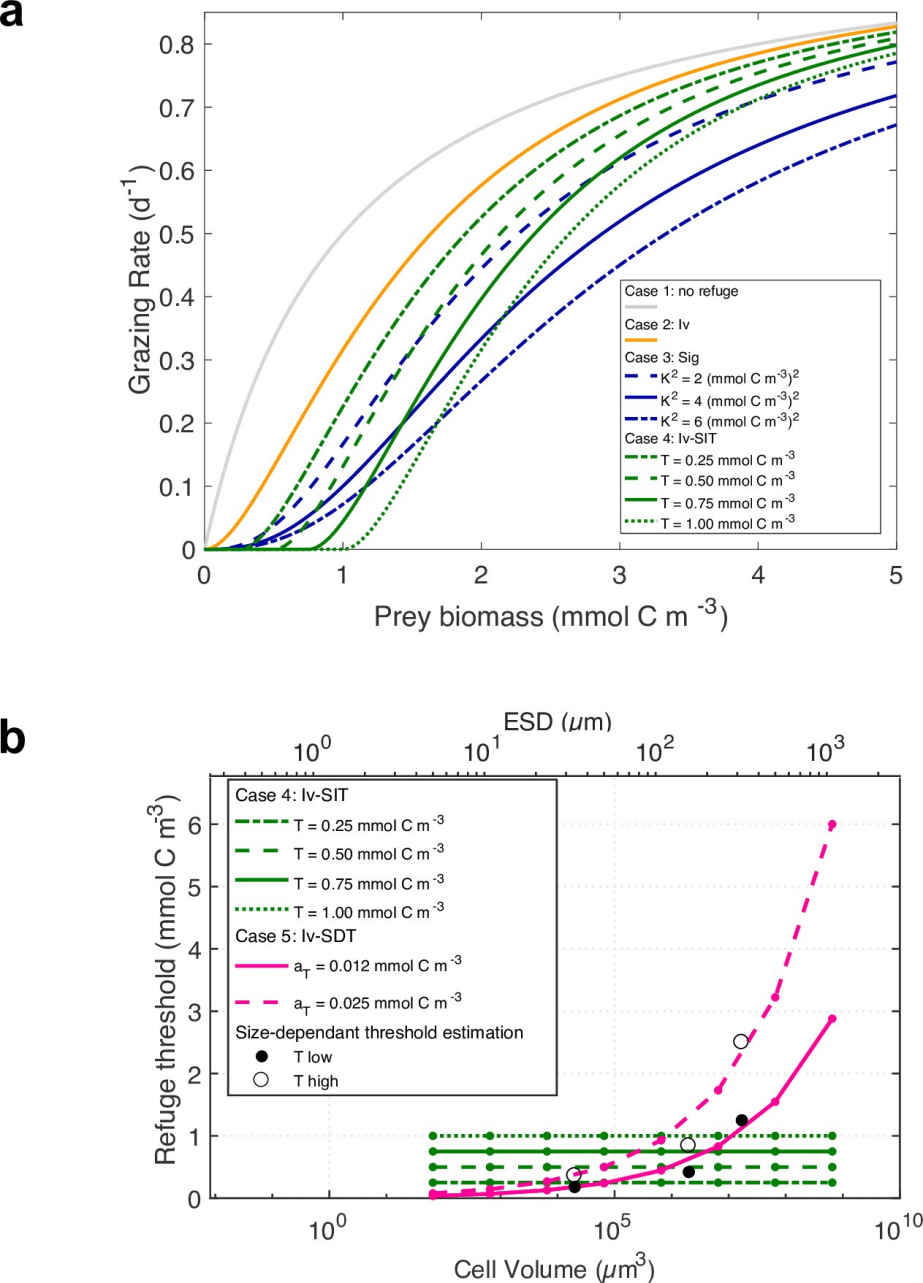
(a) Grazing effort as a function of prey biomass for cases 1–4 from Table 1. Solid lines correspond to cases presented in the core paper (other cases are presented in supporting information). (b) Specification of an explicit refuge food concentration T where grazing is initiated for case 4 (size-independent threshold, green lines with four different parameter values) and case 5 (body size-dependent threshold T as a function of zooplankton biovolume and ESD, pink lines). Black and white dots correspond to low and high estimates from the literature, respectively; pink lines represent T fitted to these estimates with an allometric relationship T = a_T_V^b^, with a_T_-low = 0.012 mmol C m^-3^, a_T_-high 0.025 mmol C m^-3^, and b = 0.27 (cases 5, Iv-SDT). Green lines represent size-independent T (cases 4, Iv-SIT).

The specific grazing rate *G* of a predator *jpred* on a prey *jprey* is given by:

$$G_{jpred,jprey}=G_{jpred}^{max}∙R∙\Phi_{\left(P\;or\;Z\right)}$$ (2)

where $G_{jpred}^{max}$ is the maximum grazing rate on prey *jprey* (referenced as the ingestion rate in Ward et al. [24]), *Φ* is the prey biomass (which can be phytoplankton *P* or zooplankton *Z*); *R* describes the prey refuge as a function of the prey availability and reduces the grazing effort as prey biomass becomes scarce: this function typically has a sigmoidal shape. Note that although we illustrate Eq (2) in terms of a single prey item, all models include multiple prey classes and the thresholds refer to summed concentration of all suitable prey combined, weighted by the size preference function described above. In this study, we evaluate the sensitivity to the specific formulation of the prey refuge by modifying the shape function *R* as described in Table 1 and represented in Fig 2 (Note that, for clarity, Table 1 illustrates the prey refuge for a single prey. The more general formulation is given in S1 Table). We consider five different cases:

- In ***case 1*** *(No refuge)*, we investigate how removing all forms of prey refuge, using only the rectangular hyperbola formulation for grazing passing through (0,0), shapes resulting ecosystem structure. In all subsequent cases, a grazing refuge is introduced by a function applied to the rectangular hyperbola in case 1.
- In ***case 2*** *(Iv)*, we test the original version of the prey refuge from QUOTA that multiplies the rectangular hyperbola by an Ivlev [54] function as done by Ward et al. [24].
- In ***case 3*** (*Sig*), we replace the Ivlev reduction by a sigmoidal function as an alternative representation of a prey refuge.
- In ***case 4*** *(Iv-SIT*, for Ivlev Size-Independent Threshold*)*, we add an explicit prey refuge to the Ivlev reduction from case 2 with a fixed, size-independent threshold prey concentration *T* [39] (see Fig 2B, horizontal lines).
- In ***case* 5** *(Iv-SDT*, for Ivlev Size-Dependent Threshold*)*, we introduce a threshold prey concentration *T* that varies with the body size of the grazer (Fig 2B, curved lines).

**Table 1:** Various mathematical formulations of the grazing rate R.

Case	Grazing refuge function	R(X) mathematical formulation	parameters to set	values	units
**1**	No refuge	$R(X)=\frac{X}{X+k_{jpred}}$	-	-	-
**2**	Ivlev (Iv)	$R(X)=\frac{X}{X+k_{jpred}}(1-e^{\Lambda X})$	*Λ*	-1	-
3a	Sigmoid (Sig)	$R(X)=\frac{X}{X+k_{jpred}}\frac{X^{2}}{X^{2}+K^{2}}$	*K*^2^	2	(mmol C m^-3^)^2^
**3b**				4	
3c				6	
4a	Ivlev Size-Independent-Threshold (Iv-SIT)	$R(x)=\{\frac{X-T}{(X-T)+k_{jpred}}(1-e^{\Lambda (X-T)}),X>T0,X\leq T$	*T*	0.25	mmol C m^-3^
4b				0.50	
**4c**				0.75	
4d				1.00	
**5a**	Ivlev Size-Dependent-Threshold (Iv-SDT)	$R(x)=\{\frac{X-T}{(X-T)+k_{jpred}}(1-e^{\Lambda (X-T)}),X>T0,X\leq T$ with $T=a_{T}V^{b_{T}}$	*a*_*T*_,*b*_*T*_	0.012, 0.27	mmol C m^-3^, -
**5b**				0.025, 0.27	

For clarity, R(X) is illustrated for a single prey X given in biomass (mmol C m^-3^), although all models include the same multiple prey size classes. Bold numbers correspond to cases presented in the core paper (other cases are presented in supporting information). Λ is the rate at which saturation of the ingestion is achieved with increasing food level. T is the zooplankton feeding threshold. a_T_ and b_T_ represent allometric parameters for the size-dependent feeding threshold.

It should be noted that *cases 2–5* utilize variations of Ivlev or sigmoidal functions only as alternative mathematical means to reduce grazing effort at low prey concentrations and are not meant to correspond to a mechanistic description of prey encounter or feeding effort. These formulations of the prey refuge *R* in Eq (2) do not correspond to the classical functional response curves (e.g., [39]).

Feeding thresholds have been defined differently in the literature, depending on whether a grazer’s ingestion rate or clearance rate is considered. For ingestion rates, a threshold is considered the lowest prey concentration at which positive ingestion is measured (e.g., *T*_*i*_ in S1A Fig). Examination of the experimental literature shows a broad range of numerical values for such a threshold, with a mean of 1.6, minimum of 0.2 and maximum of 2.6 mmol C m^-3^ for copepods and other crustaceans, ciliates, and heterotrophic dinoflagellates [55–69].

In the context of clearance rates, a threshold is a prey concentration at which clearance rate (i.e., volume swept clear of prey predator^-1^ time^-1^) decreases below its maximum, as prey concentrations decline (*T*_*c*_ in S1B Fig). From the same literature sources as above, such thresholds show a mean of 2.4, a minimum of 0.4 and a maximum of 5.0 mmolC m^-3^. This broad range of measured values leads us to consider two alternative formulations for feeding thresholds. In the first, the grazing threshold is held constant for all size classes of zooplankton (case 4, illustrated in Fig 2B). Different values for this fixed threshold are considered, ranging from 0.25 to 1.0 mmolC m^-3^ (Fig 2A and 2B). In the second, the grazing threshold increases with increasing body size of the grazer (case 5a and 5b, Fig 2B). For case 5, we treat an example of low body size-dependence (i.e., the threshold varies from 0.04 to 2.9 mmolC m^-3^) and high body size-dependence (threshold from 0.08 to 6.0 mmolC m^-3^) across consumer biovolumes ranging from 10^2^ to 10^9^ μm^3^ (Fig 2B).

In cases 3 and 4, we test the sensitivity to the prey refuge parameters. However, for simplicity, we choose to present only one of the three and four experiments performed in ***cases 3*** and ***4***, respectively, in the core paper: case 3b and 4c (with sensitivity tests results provided in supporting information).

### Metrics for quantifying ecosystem structure and response to grazing formulation

#### Chl-a spatial distribution

As a first index of the ecosystem model response to grazing formulation, we choose Chl-*a* annual climatological mean concentration. The model Chl-*a* is compared to satellite and in situ data. We use climatological SeaWiFS (Sea-Viewing Wide Field-of-view Sensor) data averaged over 1997–2005. We also examine the vertical and cross-shore structure of the model using CalCOFI in situ chlorophyll-*a* climatologies (https://calcofi.org/) averaged over 1984–2000, along line 70 (south of Monterey, California), from the coast to 350 km offshore. Climatologies of total phytoplankton and zooplankton carbon biomass were obtained from data reported in Kenitz et al. [70], along CalCOFI line 90 for the period Nov. 2004-Dec. 2010 for phytoplankton and Nov. 2005 –Jan. 2011 for mesozooplankton. Phytoplankton were sampled at 3 depths in the euphotic zone by water bottles and analyzed by flow cytometry and epifluorescence microscopy, then each taxon converted to organic carbon from literature regressions (see [70,71] for details) and vertically integrated from the deepest sampling depth to the surface. Unlike Kenitz et al. [70], we utilize all 3 sampling depths. For microplankton taxa known to include mixotrophs, half of the biomass was assigned to phytoplankton and half to zooplankton carbon. Mesozooplankton were sampled by a vertical PRPOOS net (Planktonic Rate Processes in Oligotrophic Ocean Systems: 0.5 m diameter, 202 μm mesh) from 210–0 m, preserved in 1.8% buffered formaldehyde, Zooscanned [72,73], converted to organic carbon from body length-to-carbon regressions [74], and vertically integrated over the upper 210 m.

#### Ecosystem structure and function

We then examine the response of the planktonic ecosystem in terms of phytoplankton functional types and both phytoplankton and zooplankton size classes. In each case, we consider that a type or size class emerges if its biomass exceeds a threshold of 10^−5^ mmol C m^-3^ on each cell grid. We compute the Z:P carbon biomass ratio, which provides insight into the structure and function of the plankton ecosystem.

We consider the seasonal variation of biomasses and grazing fluxes in order to diagnose the emergence of community succession and understand the effect of grazing pressure on plankton ecosystem structure. The seasonal development of the different quantities (biomasses, fluxes, Z:P ratio) is computed over the full depth, excluding up to 450 km (30 grid points) at the edge of the modeled domain to remove noisy signals generated by boundary forcing.

#### Spatial diversity and niches

The model permits the coexistence of different plankton types in the same location [24]. To diagnose such coexistence, we use species richness. We define species richness as the number of phytoplankton size classes of each pft, or number of zooplankton size classes, given by the biomass concentration *Bj*, that exceeds a relative threshold of *Bj*>10^−5^ *max*(*Bj*) [26].

Environmental heterogeneity of physical properties creates a variety of ecological conditions that promote different habitats favoring different plankton types. In addition to these environmental factors, functional traits of species–such as resource uptake and grazing–shape different habitats or niches. From our model outputs, we defined niches as the geographical extent where the biomass of a phytoplankton size class of each pft, or zooplankton size class, dominates. Both richness and niches are computed from annual mean biomass from the 6-year averages.

## Results

### Chl-*a* spatial distribution

**Fig 3:**
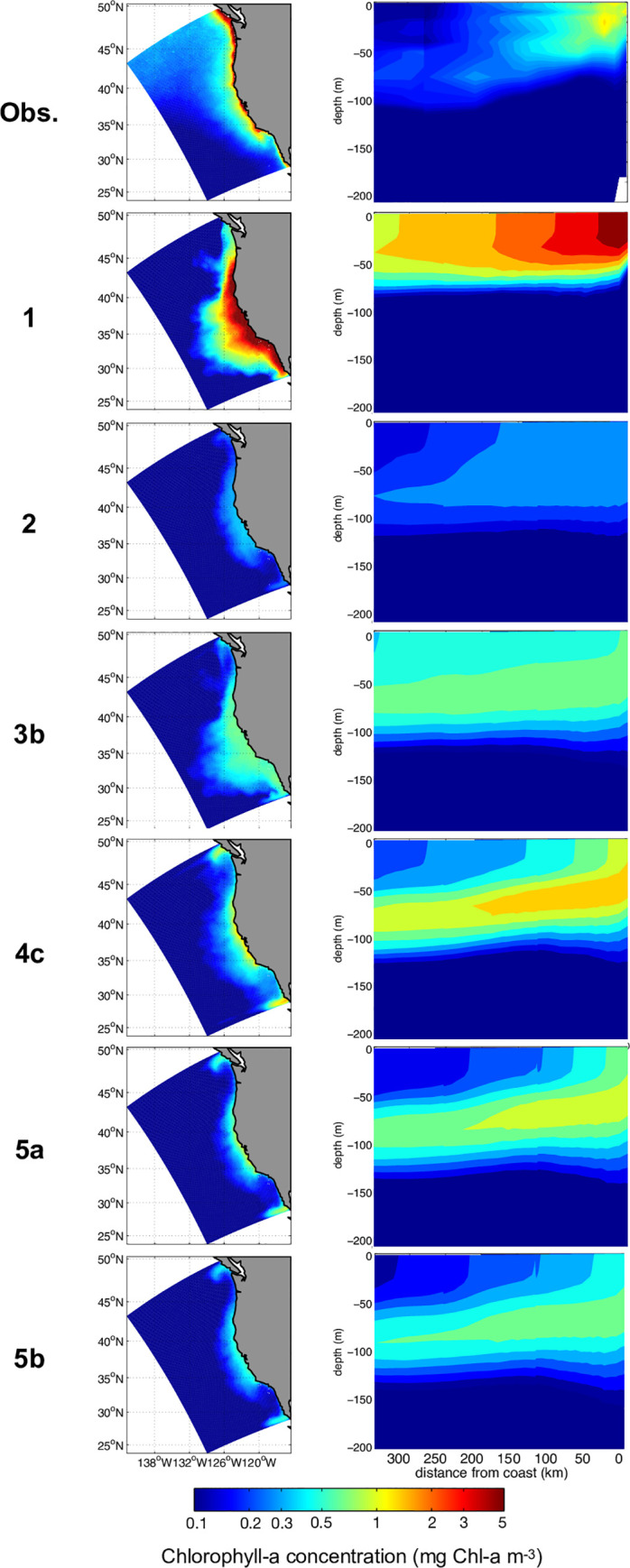
(column 1) Annual mean surface chlorophyll-a concentration (mg Chl-a m^-3^); the top panel corresponds to observations from SeaWiFS, while other panels correspond to model outputs for grazing cases 1 to 5b. (column 2) Vertical sections of annual mean chlorophyll-a concentration across CalCOFI line 70 (mg Chl-a m^-3^); the top panel corresponds to observations from CalCOFI, while other panels correspond to model outputs at the same location for cases 1 to 5b.

The annual mean chlorophyll concentration across the California Current System from SeaWiFS satellite observations (Fig 3 “Obs.”, column 1) shows the expected band of elevated Chl-*a* near the coast, sharp lateral gradients in the offshore direction, and a diminution in waters south of Pt. Conception (~34°N). The vertical section of mean Chl-*a* measurements from CalCOFI line 70 (~35.5°N, Fig 3 “Obs.”, column 2), just south of Monterey Bay, shows a nearshore surface maximum, with decreasing concentrations and the standard deep Chl-*a* maximum (DCM) in the offshore at 70–80 m depth. The five model cases with different grazing formulations differ markedly in relation to these basic geographic and vertical patterns (Fig 3). Cases 1 (*no refuge*) and 2 (*Iv*) present the largest disagreement with observations, resulting in pronounced overestimation and underestimation of chlorophyll-*a*, respectively. These biases are apparent in both the offshore and southern extent of elevated Chl-*a* concentrations seen in plan view and misrepresentation of the vertical Chl-*a* structure in section view (Fig 3.1 and 3.2, column 1 & 2). In contrast, modeled chlorophyll concentrations from grazing cases 3 (*Sig*), 4 (*Iv-SIT*) and 5 (*Iv-SDT)* show less pronounced difference in relation to observations from SeaWiFS and CalCOFI (Fig 3.3b, 3.4c, 3.5a and 3.5b, columns 1 & 2). The cross-shore gradient is somewhat better represented, with elevated concentrations at the coast extending to lower concentrations in offshore oligotrophic waters, and a DCM at about 70–80 m depth. We wish to underscore that the only variation among these different cases is altered grazing formulations.

### Ecosystem structure and seasonal cycle

Among the 22 plankton compartments initialized in the model (including both size and pft), 14 to 18 plankton compartments (i.e., 64 to 82% of initialized plankton compartments) can emerge and persist, depending on the grazing dynamics. The two largest *diatoms* (D4 and D5) and the two largest *small eukaryotes* (E4 and E5) never persist, except in case 2 where E4 faintly emerges (concentrations <10^−7^ mmol C m^-3^ on average; details not shown).

**Fig 4:**
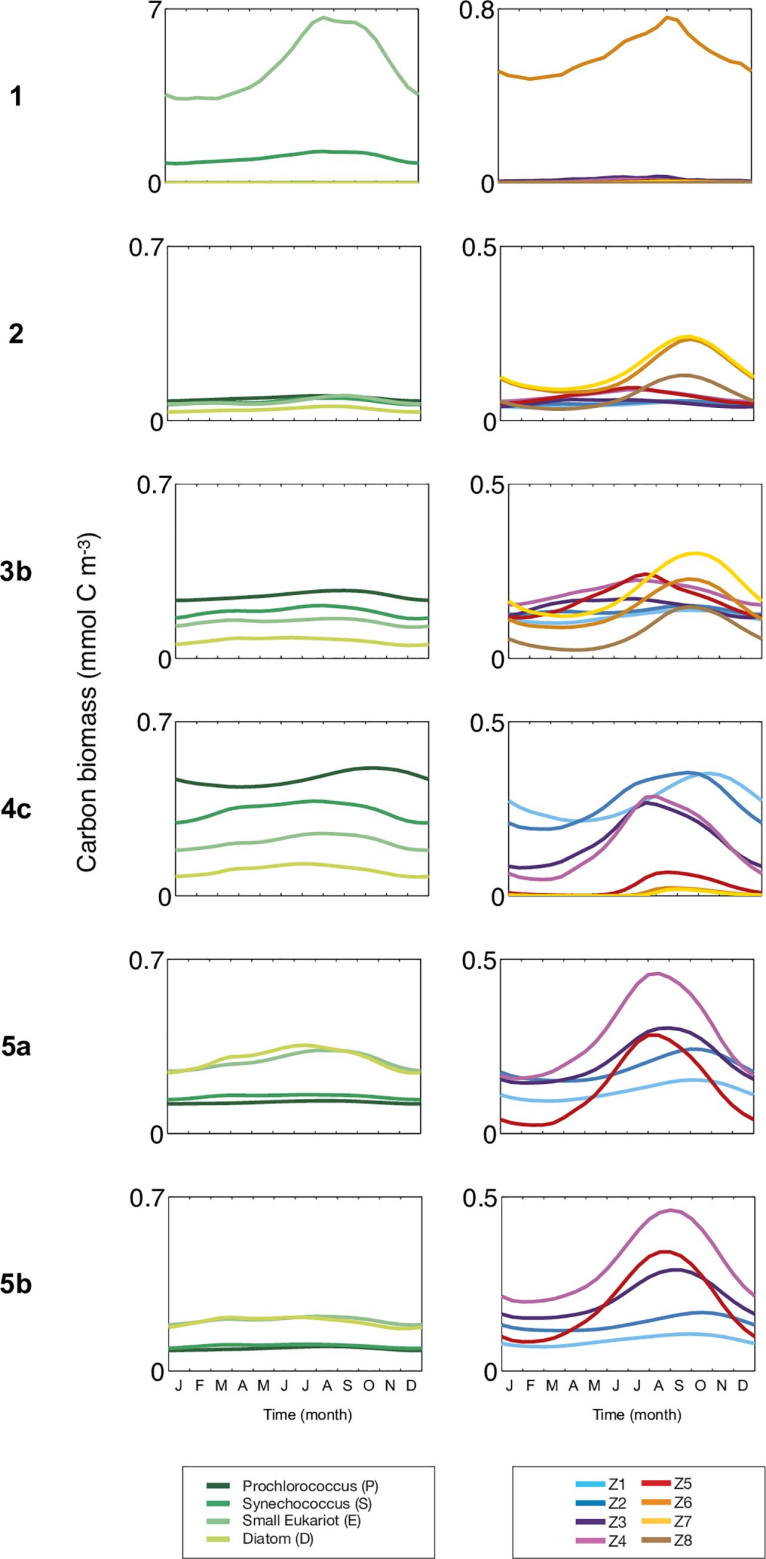
Seasonal variation of (column 1) the 4 plankton functional types (mmol C m^-3^) and (column 2) detailed size classes of zooplankton (mmol C m^-3^).

A seasonal cycle of biomass of varying amplitude occurs in all grazing cases, but maxima can vary 20-fold depending on the grazing formulation (compare phytoplankton concentrations in Fig 4 between cases 1 and 2; note the change in scale). In terms of phytoplankton dominance, in case 2, *Prochlorococcus* is the most abundant pft except in September, when *small eukaryotes* have a slight advantage. Note that in case 1 *diatoms* barely occur, and in cases 2–4 *diatoms* have the smallest biomass (S2 Table, S2 Fig). In contrast with case 2, total phytoplankton biomass is higher in cases 3 and 4 by a factor of 2 and 4, respectively (Figs 3 and 4). The relative dominances of phytoplankton pfts are similar between cases 3 and 4: all year, *Prochlorococcus* biomass is higher than *Synechococcus* biomass, followed by the biomass of *small eukaryotes* and then *diatoms*. Lastly, in both cases 5a and 5b, the dominant biomass shifts markedly to large size classes of *diatoms* and *small eukaryotes*, which have twice the biomass of *Synechococcus* and *Prochlorococcus*. Among the dominant pfts, *diatoms* can dominate during spring, while *small eukaryotes* outcompete *diatoms* during fall and winter (Fig 4).

**Fig 5:**
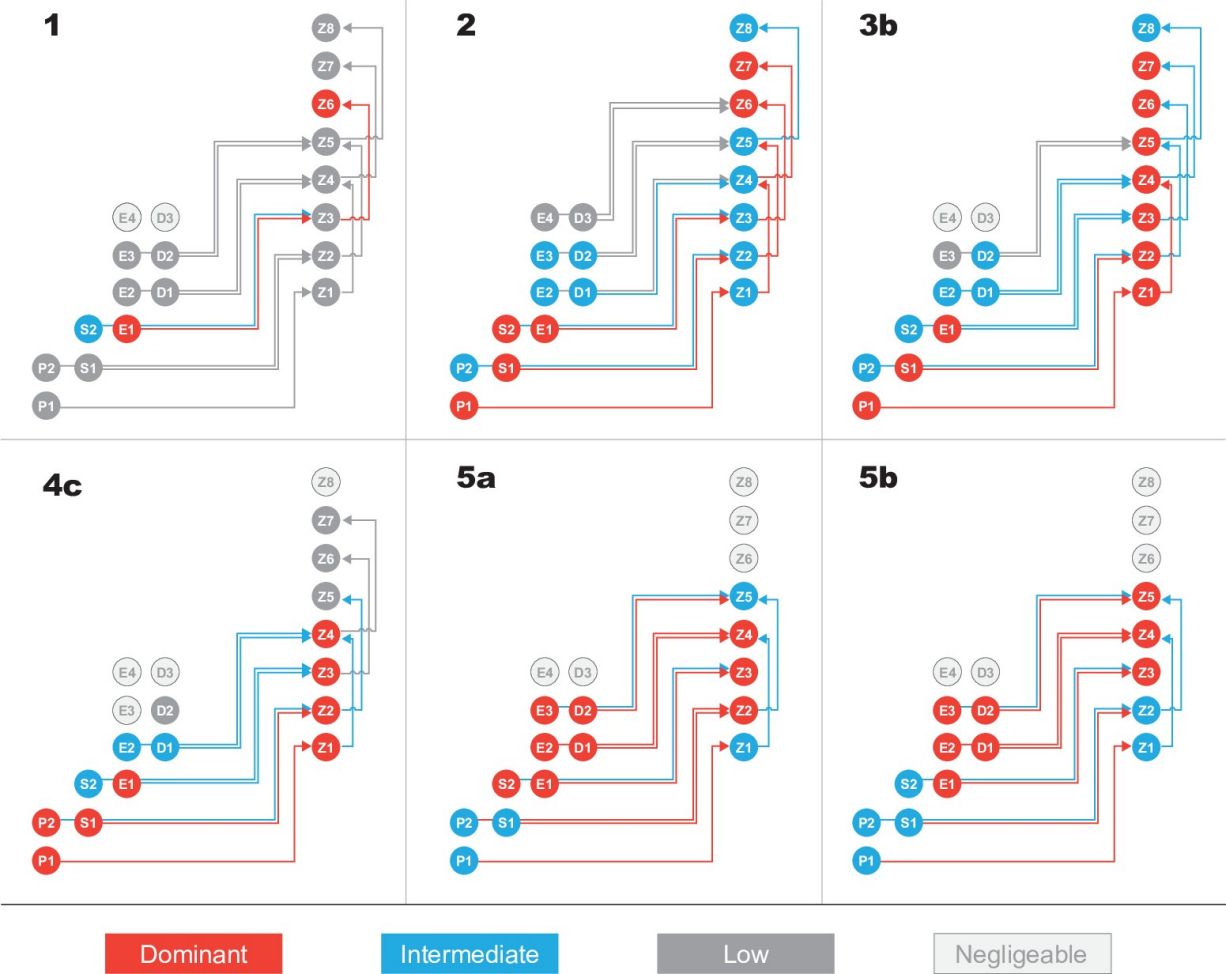
Simplified schematic representation of grazing and predation fluxes in the ecosystem model for grazing cases 1 to 5b. For each case, biomasses (circles) and fluxes (arrows) are color-coded as follows: Dominant in red, intermediate in blue, and low in dark grey. Negligible fluxes are not shown, and negligible biomasses are represented in light grey. See S1 and S2 Tables for detailed biomasses and grazing fluxes, respectively.

In terms of phytoplankton size structure, for all cases and within each pft, the amplitude of biomass variation decreases inversely with size, with the two smallest size classes emerging systematically as dominants (Fig 5, S2 Table, S2 Fig). The bigger size classes of *small eukaryotes* and *diatoms* can emerge only in certain grazing pressure conditions that include explicit refugia (see below), which allow significant emergence of 5 to 8 size classes of zooplankton (Figs 4 and 5). Despite relatively small differences in emergence of phytoplankton size classes (S2 Fig), the zooplankton size structure varies greatly among cases (Fig 4). In cases 1 and 2, large zooplankton (mesozooplankton-like, Z6, Z7, or Z8) dominate throughout the year; in case 3 there is a succession of dominance between microzooplankton during spring, and larger zooplankton during summer and fall. In all subsequent cases, there is a shift to smaller zooplankton size classes. In cases 4c and 4d, the smallest zooplankton (nanozooplankton) dominate all year long (Fig 4 and S3 Fig), or alternate with mid-sized zooplankton in cases 4a and 4b (S3 Fig); in case 5, mid-sized zooplankton dominate. Independent of dominance, all size classes of zooplankton show a clear seasonal progression from spring to fall (Fig 4). For cases 2 to 5, zooplankton biomass reaches its maximum in September for large-sized zooplankton, and between June and August for mid-sized zooplankton. However, small-sized zooplankton biomass peaks at different dates depending on the grazing cases: in March in cases 2 and 3, in June in case 4 and in October in case 5.

The CCS is known to present a strong cross-shore gradient of physical and biogeochemical properties, with contrasting ecosystem structure (dominated by different sizes (and pfts) of plankton). In order to check whether the ecosystem was sensitive to the cross-shore gradient of environmental conditions, we performed similar diagnostics separately on nearshore and offshore regions for the Southern CCS (S8–S10 Figs for phytoplankton and zooplankton, respectively). For all experiments, in terms of concentrations and seasonal dynamics, we find that the ecosystem responds positively to coastal nutrient input from the coastal upwelling with earlier and higher production compared to the offshore regions. However, in both regions we find similar sensitivity to the formulation of grazing refugia as we observe for the average over the entire CCS (Fig 4).

### Trophic interactions and variability of grazing fluxes

In all cases, grazing fluxes show a clear seasonal cycle in phase with the temporal evolution of zooplankton biomass (S4 Fig), and grazing on phytoplankton dominates over predation on zooplankton by a factor of about 2. Here, we mainly discuss the spatio-temporal average of trophic interactions, as illustrated in Fig 5, which summarizes emergent compartments and grazing fluxes (see S4 Fig for detailed seasonal cycles):

- Case 1 is a peculiar case highlighting emergence of a simple trophic assemblage with only three primary compartments: two phytoplankton size classes (E1 and less importantly S2) and one zooplankton size class (Z6; see Fig 5.1). Note that Z3 emerges weakly but represents a key compartment because it involves fluxes of carbon: grazing of E1 by Z3 and predation on Z3 by Z6. Grazing of S2 by Z3 is one order of magnitude lower. It also reveals that the emergence of large zooplankton (Z6) is permitted by predation on small zooplankton (Z3).

In contrast, all other cases present a more complex response of the plankton ecosystem characterized by a large number of significant grazing and predation fluxes (more than ten):

- In case 2, four phytoplankton size classes (P1, S1, S2, E1) and two zooplankton size classes (Z6 and Z7) dominate the ecosystem. However, intermediate to high fluxes through consumers involve all the zooplankton size classes from Z1 to Z6 (dominant predation fluxes in Fig 5). Even though Z1 to Z5 do not dominate the ecosystem in terms of biomass, they play a key role in ecosystem structure. Indeed, the smallest zooplankton (Z1 to Z5) (i) grazes on the smallest size classes of *Prochlorococcus*, *Synechococcus*, and *small eukaryotes* and (ii) are predated by large zooplankton, which can then emerge.
- Case 3b shifts to a broader spectrum of co-occurring zooplankton, with dominance of zooplankton from small Z1 to large Z7, and only three phytoplankton compartments of small size (the smallest size classes of *Prochlorococcus*, *Synechococcus*, and *small eukaryotes*). The primary fluxes involve grazing of small-sized organisms (grazing of P1 by Z1, of P2 by Z2) and predation of Z1 by Z4. Even though grazing of larger phytoplankton size classes (*small eukaryotes* and *diatoms*) and predation of mid-size zooplankton are low, they are not negligible, showing that the functioning of the ecosystem involves a large number of fluxes (N = 12).
- In case 4c, emergence of larger zooplankton size classes is inhibited, limiting zooplankton diversity. Small size classes of zooplankton and phytoplankton dominate the ecosystem (Z1 to Z4, and P1, P2, S1, and E1, respectively), with a reduced number of fluxes (8).
- Case 5a contrasts with all other cases: ecosystem structure is dominated by large phytoplankton (S2, E1, E2, E3, D1, and D2) and by small and mid-sized zooplankton (Z2, Z3, and Z4). Notably, this is the first case where any size class of *diatoms* emerges as a dominant phytoplankton leading to major carbon flux. There are more dominant grazing fluxes than in other cases, and they involve grazing by zooplankton Z1 to Z5 on phytoplankton P1 to D2. Predation terms are significant and concern fluxes from Z1, Z2, to Z4, Z5, respectively. In this case, the functioning of the ecosystem is more complex than in preceding ones, with numerous grazing and predation fluxes (ten in total). Case 5b is similar to case 5a with a slight shift to larger size classes for phytoplankton (S2 is no longer dominant), significant diatom fluxes (D1 and D1), and larger zooplankton (Z2 is not dominant anymore while Z5 turns to dominant).

**Fig 6:**
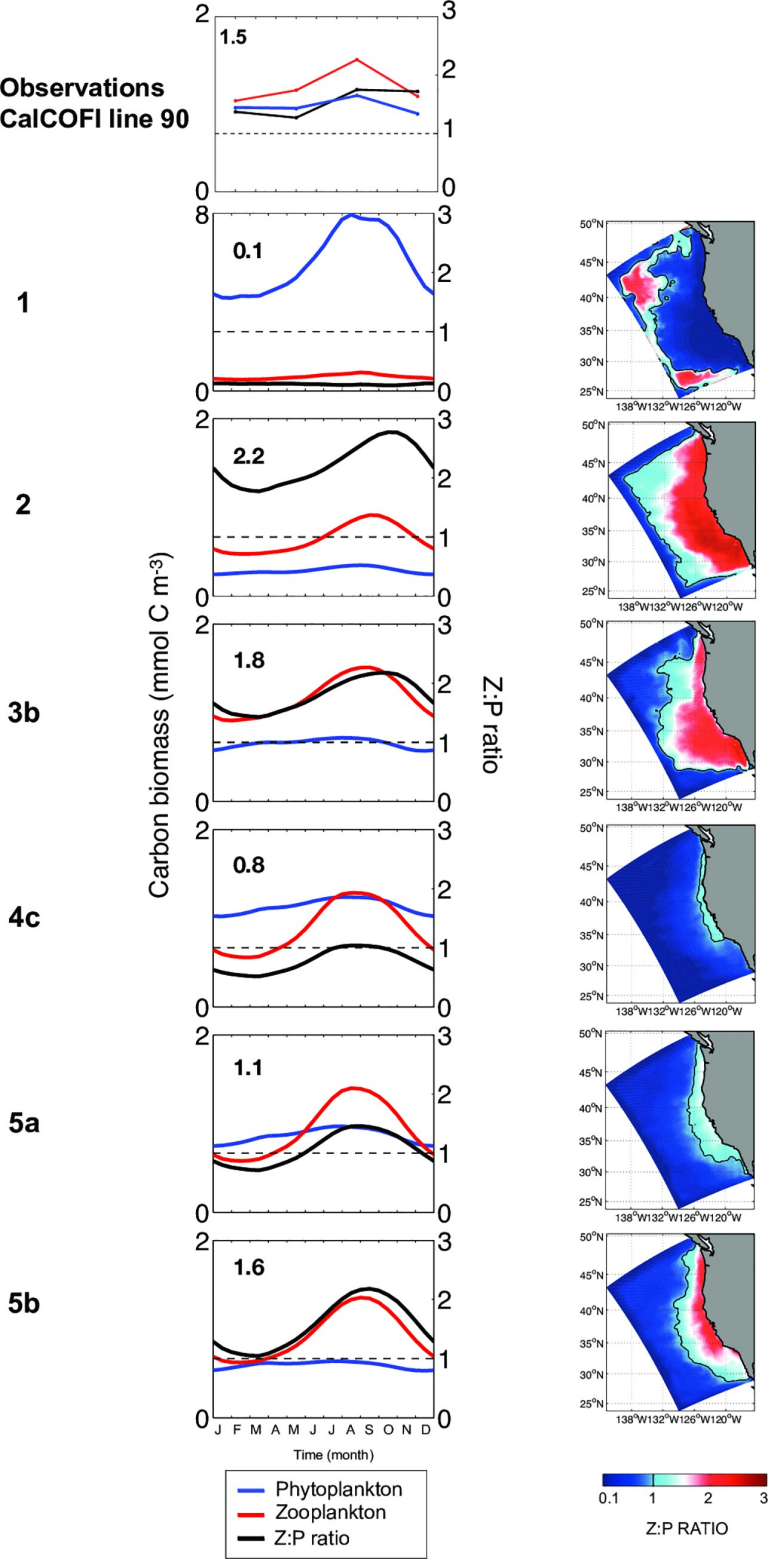
Z:P ratio. (column 1) Seasonal variation of total phytoplankton biomass (blue), total zooplankton biomass (red) and the associated Z:P ratio (black), in mmolC m^-3^. The top panel corresponds to observations from CalCOFI line 90, while other panels correspond to model outputs for grazing cases 1 to 5b. The number in the upper-left corner indicates the seasonal mean of the Z:P ratio. (column 2) Spatial distribution of the annual-mean Z:P ratio. The solid black line indicates Z:P = 1.

The relative zooplankton and phytoplankton biomass can be assessed with Z:P ratio (Fig 6) and compared with in situ observations from CalCOFI line 90 (33.485°N, 117.768°W to 30.418°N, 123.999°W) in the southern part of our study domain (Fig 6 “Observations” in top row). In the peculiar case 1, with no grazing refuge of any kind, Z:P ratio is very low (0.1) all year long and over the entire domain, suggesting weak grazing control of primary production and marked departure from observations. For cases 2 to 5, Z:P ratios show a seasonal cycle with variable relative values: in cases 2 and 3b, the Z:P ratio is high (~2) and consistently exceeds observations. In case 4c the Z:P ratio is low (= 0.8). In cases 5, the Z:P ratio is increased compared to case 4, resulting in an intermediate Z:P ratio and seasonal cycle that provides somewhat better agreement with observations. The difference between parameter values in 5a and 5b results in a higher Z:P ratio in case 5b, with an overall seasonal mean of 1.6, which compares with a value of 1.5 from observations (Fig 6). From a spatial point of view, all cases (except case 1) present a cross-shore gradient with higher Z:P at the coast, but the cases differ substantially in the spatial extent of cross-shore extension of Z:P ≥ 1, which varies from ~100 km to 1000 km offshore.

Again, we check whether the total biomasses and Z:P ratio are sensitive to the cross-shore gradient of environmental conditions (S10 Fig). We find that total phytoplankton and zooplankton biomasses averaged over the nearshore and offshore areas for the Southern CCS respond coherently to the cross-shore gradient with higher concentrations inshore in response to higher concentrations of upwelled nutrients. Apart from the extreme case 1, the nearshore and offshore regions exhibit similar sensitivities to grazing dynamics.

### Spatial niches and richness

**Fig 7:**
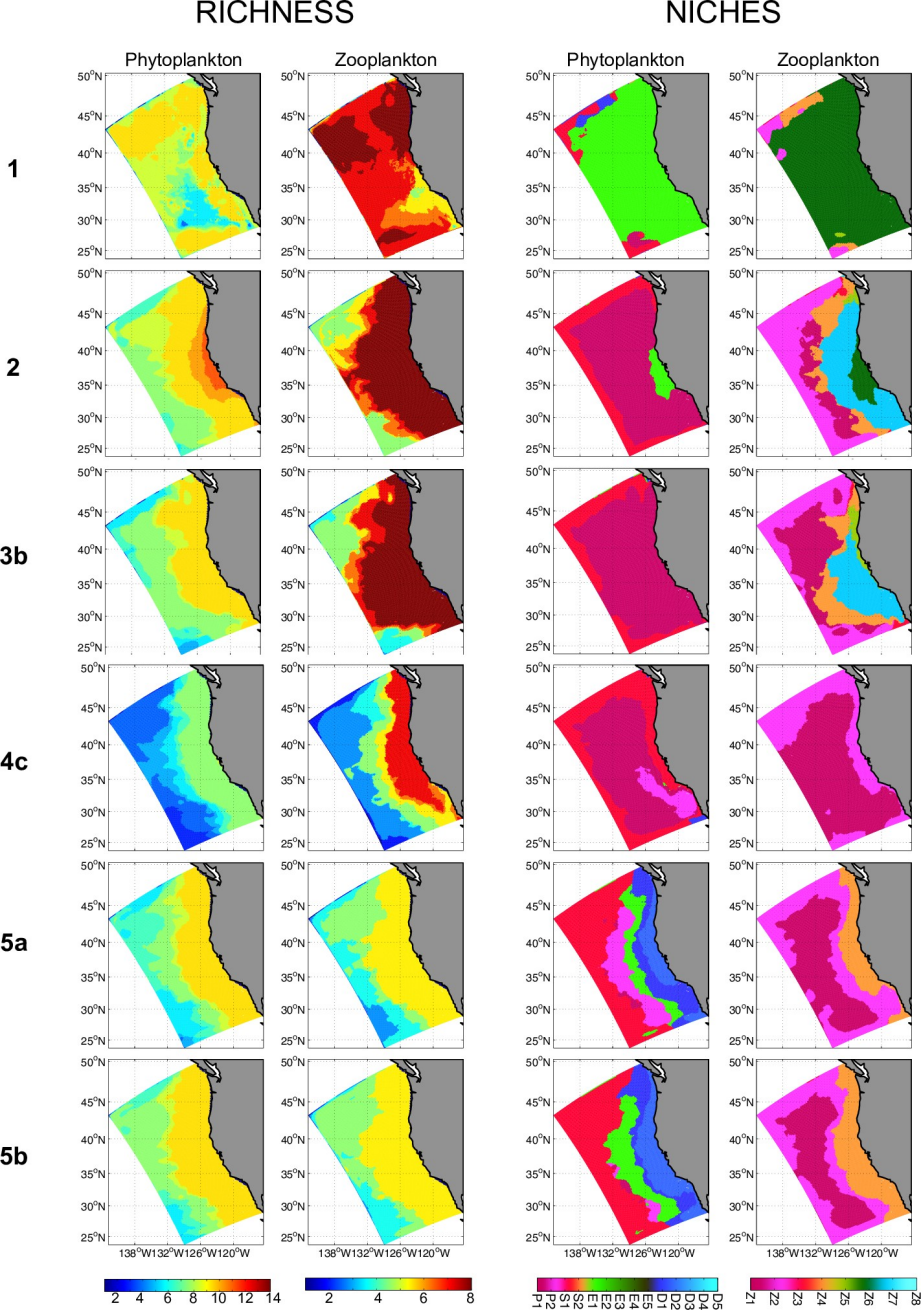
(columns 1 and 2) Richness and (column 3 and 4) niches of phytoplankton (columns 1 and 3) and zooplankton (columns 2 and 4) for grazing in cases 1 to 5b.

Both total species richness and the spatial niches occupied by different plankton categories are highly sensitive to the zooplankton grazing formulation used. In the peculiar case 1, in terms of equilibrium richness, up to 9 phytoplankton compartments and 8 zooplankton compartments emerge (Fig 7, top row of columns 1 & 2), while niches are overwhelmingly dominated by only E1 and Z6 over the entire CCS (Fig 7, top row of columns 3 & 4).

In all other cases, richness decreases in the cross-shore direction, with no clear latitudinal trend (Fig 7, column 1 & 2). Species richness varies among cases, with highest richness for case 2 for both phytoplankton and zooplankton, lowest phytoplankton richness in case 4, and lowest zooplankton richness in case 5. Except in case 1, niches show a cross-shore gradient, with a coastal dominance of larger size classes for both phytoplankton and zooplankton (Fig 7, column 3 & 4). In case 2, small species niches dominate the domain with E1 in coastal waters (exclusively in the central CCS), and P1 elsewhere; despite few phytoplankton niches, there is a high diversity of zooplankton niches with large size classes (Z6 and Z7) at the coast and small ones offshore (Z1 and Z2). Case 3 presents a single dominant phytoplankton niche (P1). Despite low diversity of phytoplankton niches in both cases 2 and 3b, these cases present a high diversity of zooplankton niches (Z5 and Z7 at the coast, Z1 and Z4 offshore). In case 4, niche diversity of phytoplankton slightly increases, while niche diversity of zooplankton is the lowest among all cases. In contrast, the simulation presenting the most diverse type of phytoplankton niches is case 5a, with—from coast to offshore water—diatoms (D2 close to the coast and D1), small eukaryotes (E1), *Prochlorococcus* (P1) and *Synechococcus* (S1). Notably, not until case 5, which incorporates a body-size dependent grazing threshold, do *diatoms* dominate in any part of the CCS. Despite such diversity of phytoplankton niches, zooplankton niche diversity is moderate, with niches of small zooplankton (nano and micro) dominating at the coast (niches of Z4, Z2, and Z1 from the coast to offshore).

## Discussion

This analysis illustrates that a suite of properties relevant to ocean biogeochemistry, pelagic food web structure, and carbon flow pathways are highly sensitive to the formulation of grazing in a contemporary planktonic ecosystem model. The model structure incorporates multiple forms of dissolved nutrients, intracellular nutrient quotas, four phytoplankton functional types, and a spectrum of size classes of both phyto- and zooplankton, with allometrically scaled parameters. The food web model is coupled to a regional ocean model (ROMS-CROCO) of the California Current coastal upwelling ecosystem, where spatial gradients are high in both the cross-shore and vertical dimensions. The model results show that changes in the functional form of grazing that are all within the scope of previous measurements can have substantial consequences for spatial patterns of plankton concentrations, species richness, food web pathways, and underlying seasonal cycles.

Our results are not unexpected, in light of the previous numerical experiments [41,42, and others]. Our findings also reinforce the results of Anderson et al. [27], who in a global ocean circulation model found high sensitivity of phytoplankton functional group distributions and biomass to the choice of grazer functional responses. However, that study did not specifically explore the importance of prey refugia or vertical distributions and considered only two zooplankton classes.

Here we discuss the results of our sensitivity analyses with increasing complexity of the grazing formulation, from ignoring refugia to implementation of a size-dependent grazing refuge. Our principal objective was not to tune parameters to most closely resemble observations but to understand the sensitivity to the form of grazing considered and provide guidance on preferred formulations of grazing in the future.

### No refuge = no diversity

*Case 1* eliminates all forms of grazing refuge, replacing it with a classic rectangular hyperbola that goes through the origin (0, 0). In *case 1*, model output disagreed with observations in the CCS with a substantial biomass overestimation, almost 5 times over chlorophyll-*a* observations. Not surprisingly, the formulation without grazing refuge fails to reproduce coherent ecosystem structure in our study region: the dominance of a single phyto- and zooplankton species and the prevalence of single species niche over the entire CCS differ completely from observations in such an upwelling system, with a cross-shore shift from coastal rich water to offshore oligotrophic waters typical of coastal upwelling systems [70,75]. Observing such a homogeneous pattern of niches in such a heterogeneous environment [45] highlights the importance of more realistic grazing formulations. Persistently low values of the Z:P ratio (spatially and seasonally) indicate abnormally high phytoplankton biomass over zooplankton biomass. Independently of the degree of realism of ecosystem dynamics of the CCS, withdrawing a grazing refuge leads to the emergence of a short food web that involves one of the smallest phytoplankton (*E1*) feeding one type of nanozooplankton (*Z3*) which is predated by a single type of mesozooplankton (*Z6*). The most competitive phytoplankter is not the smallest—with the highest nutrient affinity - or the biggest—with the highest growth rate—but an intermediate. In sum, in this peculiar *case 1*, by removing the grazing refuge, we introduce non-negligible grazing pressure over low-density prey that limits the emergence of an equal diversity among plankton size classes independently of environmental variability. In the HNLC (High Nutrient-Low Chlorophyll) conditions in the Equatorial Pacific, Leising et al [41] similarly could not reproduce ambient conditions without inclusion of a grazing threshold in a simpler Fe-N-P-Z model.

### Classic refuge formulations and the emergence of diversity

Empirical formulations of grazing that are typically used in modelling studies include a simple and fixed grazing refuge for prey (see [42] for references, or as in the original version of the QUOTA model from [24]). We test the sensitivity of such empirical formulation in *case 2* and *case 3*. Implementing such a refuge for low prey density with classic sigmoidal grazing formulation increases phytoplankton diversity of smaller species, i.e., *Prochlorococcus* and *Synechococcus*, as well as zooplankton diversity. This diversity mirrors complex trophic interactions with multiplicity of grazing and predation fluxes from small to large size classes. Interestingly, diversity of zooplankton is dominated by two large zooplankton in case 2, while in case 3, this diversity is homogeneous with clear successions of diverse zooplankton assemblages (small and large size classes). Such an emergence of large size classes of zooplankton is possible through high predation pressure. Smaller-sized zooplankton do not dominate in case 2 because of high fluxes through this compartment (from grazers to their predators). As discussed in Ward et al. [24], a model that explicitly resolves the grazer community allows the “kill-the-winner” mechanism to maintain phytoplankton diversity, according to Armstrong [76]. However, our results reveal that such diversity depends not only on the diversity of the grazers but also crucially on how they graze by introducing a classic refuge formulation.

Such classical grazing formulations allow the emergence of diversity with limitations. With an Ivlev formulation (*case 2*), biomasses are markedly underestimated compared to observations, and larger species such as diatoms do not emerge in nutrient-rich coastal water [77]. On the other hand, with a stronger suppression of grazing at low concentrations achieved by a sigmoidal formulation (*case 3*), the misfit to phytoplankton biomass and its spatial distribution is less extreme, though still highly biased. In this case, trophic interactions are modified, leaving more capacity for small size classes compared to mid classes to grow (*case 2*). The main differences between these two cases occur within zooplankton dynamics, and not within phytoplankton dynamics, with a partial reduction in predation pressure for the sigmoid formulation.

Within *case 3*, increasing the refuge (with increased values of *K*^*2*^) allows a reduction of grazing pressure for the same prey density. This promotes an increase of both phytoplankton and zooplankton biomass, especially emergence of small phytoplankton and nanozooplankton earlier in the year and a decrease of the Z:P ratio.

Simple refuge formulations as in cases 2 and 3 are typically used because of the lack of data [42], while it is now clear that such a grazing formulation in a continuum size-structured ecosystem model leads to substantial inaccuracies.

### Toward an explicit threshold

Lastly, we implement an explicit threshold prey concentration below which grazing is zero, as either a fixed value for all size classes (*case 4)* or a refuge that increases as the consumer size increases (c*ase 5*). In both *cases 4* and *5*, trophic interactions are still quite complex with various emergences of pfts and size classes. We recognize that Leising et al. [41], as expanded on more fully in Strom et al. [78], illustrate how the feeding behavior of individual grazer types, each with different feeding thresholds, can appear in the aggregate to exhibit no threshold behavior.

A body size-independent grazing refuge threshold (*case 4*) increases phytoplankton and zooplankton biomass to somewhat more realistic levels and shifts plankton composition towards smaller size classes compared to other cases; it also allows an increase in phytoplankton niches and a decrease in zooplankton niches. Increasing the threshold refuge *T* (*case*s *4*) enhances phytoplankton biomass (S5 and S6 Figs) through less top-down control (i.e., less grazing pressure through less zooplankton biomass, (S6 Fig)) but decreases both niche diversity and richness for phyto- and zooplankton (S7 Fig).

Implementing a size-dependent refuge threshold (case 5) allows significant changes in plankton dynamics: (i) there is a somewhat better agreement of phytoplankton biomass with observations and (ii) plankton ecosystem structure is more realistic for the CCS with the emergence of large phytoplankton and, notably, coastal dominance of diatoms (for *case 5a*) [77]. In this case, the larger the plankton size class is, the higher the refuge. Zooplankton diversity is relatively low and is mainly made up of smaller zooplankton (nano- and microzooplankton), involving more grazing fluxes than predation fluxes compared to other cases. There are more trophic interactions (or fluxes) at play but with lower intensity. Altered parameter values within case 5 mainly affect phytoplankton, permitting larger organisms to emerge. The steeper body size-dependent thresholds enable both small and large organisms to escape grazing and finally allow diatoms to dominate in the ecosystem, promoting diversity (i.e., in terms of richness and diversity of niches). This leads to higher carbon transfer into the food web as soon as the bloom of diatoms appears (higher Z:P ratio), and permits more realistic Z:P ratios in relation to in situ observations. Some evidence for a body size-dependent feeding threshold within a species comes from the results of Olivares et al. [79], whose study suggests that adult female *Paracartia grani* have appreciably higher threshold concentrations than younger developmental stages.

In another set of experiments, we tested the sensitivity of the model to the grazing kernel shape (results not shown). Increasing the range of prey up to reasonable values (i.e., increasing the normal distribution width from 0.5 to 0.915) or changing the normal distribution to an asymmetric distribution allowing grazing on smaller prey does not appreciably change our results.

We intentionally do not consider a number of processes that could be explored further because of our focus on changing only one aspect of an existing model structure. We do not consider the effects of acclimation to prey (e.g., [12,42]), variable satiation responses, departures of predator:prey size ratios from 10:1 [80], or differences in half-saturation constants for different prey types or different predators. We also recognize that, in addition to consumer body size, differences among taxa and differences in ocean habitat can affect prey thresholds. For example, Paffenhöfer and Stearns [81] suggested that copepods occurring in offshore and oceanic environments show feeding thresholds at lower food concentrations than more coastal copepods. Lessard and Murrell [82] indicated that the grazing threshold for microzooplankton in the oligotrophic waters of the Sargasso Sea corresponds to the annual minimum chlorophyll concentration found in the mixed layer. There are also suggestions that the minimum food concentration for positive net growth increases with temperature (e.g., [83]), although feeding thresholds may not be temperature-dependent [66]. We also do not consider the relative motility of different predator and prey types, turbulence-mediated modifications to encounter, prey switching responses, algal toxicity, or many other important details of planktonic predator-prey interactions [43,84]. Furthermore, the present model does not address the importance of the mathematical formulation of zooplankton mortality, a closure term that has been widely recognized to affect the dynamics of planktonic ecosystem models [42,85,86].

Our work does not seek to fully model realistic ecosystem structure and diversity over the CCS. Goebel et al. [87,88] utilized a ’’self-organizing’’ model [4] to describe diversity patterns off the California coast, although there were limited validation data available for the region. While the model we employed does include some diversity, this is only at a low level (only 14 phytoplankton and 8 zooplankton compartments) due to several constraints: the multiplicity of experiments conducted, the horizontal resolution of the model, and the available computing time. Nevertheless, such a simple size-structured ecosystem model allowed us to diagnose key processes.

## Conclusions

In conclusion, small changes in grazing refuge formulation drive large modifications in plankton diversity, structure, and ecosystem functioning. These results reiterate the importance of careful formulation of the grazing refuge to accurately represent plankton dynamics, as well as for a spectrum of ecosystem processes. This work seeks to inform future formulations of ecosystem models. While finer spatial resolution and better representation of mesoscale eddies and submesoscale filaments [46,89] are laudable goals for future models, here we emphasize the importance of biological structure and, in particular grazing processes. We show that fundamental differences in model results emerge from a simple modification of grazing refugia across a range of plausible functional forms.

Our results suggest that an explicit grazing refuge, with refuge prey concentration dependent on the body size of grazers, is likely to provide more coherent plankton ecosystem dynamics compared to classic formulations or size-independent threshold refugia. Therefore, our recommendation for future models of plankton ecosystem structure in a continuum size-structured model is to implement a size-dependent threshold refuge. As for parameter choice, we suggest use of our less steep size-dependent case (case 5a, i.e., *a*_*T*_ = 0.012 mmol C m^-3^), which appears closer to the modest available literature data. We also recommend initiation of a new suite of experimental grazing studies intended to test the hypothesis of body size-dependent grazing refugia across a broad spectrum of planktonic grazers, and to rigorously establish appropriate parameter values for future models.

One may wonder whether such sensitivity to grazing pressure would emerge in another region. In light of the environmental heterogeneity observed in the California Current Ecosystem, which extends from nutrient-rich coastal waters to offshore oligotrophic waters and encompasses most of the range of optical properties found in the world ocean [90], we suggest that these findings are likely quite general. Moreover, given the importance of Eastern Boundary Current Upwelling Ecosystems for marine biogeochemistry and trophic interactions, it is particularly important that they be more accurately represented in models that seek to represent global scale ocean processes.

## Supporting information

S1 Fig Schematic representation of (a) ingestion rate and (b) clearance rate for a classic sigmoidal functional response (solid line) vs. a response considering a feeding threshold *T*_*i*_ and *T*_*c*_, (for ingestion rate and for clearance rate, respectively) (dashed line).
(DOCX)

S2 Fig Seasonal variation of detailed pfts accounting for emergent phytoplankton size classes (in mmol C m^-3^). (DOCX)

S3 Fig Seasonal variation of (column 1) the 4 plankton functional types (mmol C m^-3^) and (column 2) detailed size classes of zooplankton (mmol C m^-3^).
(DOCX)

S4 Fig Seasonal variation of grazing fluxes (averaged on the last year of simulation, integrated over depths and averaged over longitude and latitude, excluding boundary conditions). Grazing fluxes correspond to equation 2. Color code corresponds to each zooplankton size class (as in Fig 3) while dotted-dashed, dashed and solid lines correspond to preys of increasing maximum growth and grazing rates, respectively (y-axis in mmol C m^-3^ d^-1^).
(DOCX)

S5 Fig (column 1) Annual mean surface chlorophyll-a concentration (mg Chl-a m^-3^); (column 2) Vertical sections of annual mean chlorophyll-a concentration across CalCOFI line 70 (mg Chl-a m-3). Results correspond to model outputs for grazing cases 3 and 4 not shown in the core paper.
(DOCX)

S6 Fig Z:P ratio. (column 1) Seasonal variation of total phytoplankton biomass (blue), total zooplankton biomass (red) and the associated Z:P ratio (black). The number in the upper-left corner indicates the seasonal mean of the Z:P ratio. (column 2) Spatial distribution of the annual-mean Z:P ratio. The solid black line indicates Z:P = 1. Results correspond to model outputs for grazing cases 3 and 4 not shown in the core paper.
(DOCX)

S7 Fig (columns 1 and 2) Richness and (column 3 and 4) niches of phytoplankton (columns 1 and 3) and zooplankton (columns 2 and 4) for grazing in cases 3 and 4.
(DOCX)

S8 Fig Comparison of phytoplankton concentrations between coastal (0-100km) and offshore region (300-400km), in the Southern CCS (+/- 0.5° around line 90 of CalCOFI): Seasonal variation of detailed pfts accounting for emergent phytoplankton size classes (in mmol C m^-3^). (DOCX)

S9 Fig Comparison of zooplankton concentrations between coastal (0-100km) and offshore region (300-400km), in the Southern CCS (+/- 0.5° around line 90 of CalCOFI): Seasonal variation of detailed size classes of zooplankton (mmol C m^-3^). (DOCX)

S10 Fig Comparison of Z:P ratio between coastal (0-100km) and offshore region (300-400km), in the Southern CCS (+/- 0.5° around line 90 of CalCOFI): Seasonal variation of total phytoplankton biomass (blue), total zooplankton biomass (red) and the associated Z:P ratio (black) from observations (CalCOFI data) and the different numerical experiments. The number in the upper-left corner indicat.
(DOCX)

S1 Table Various mathematical formulations of the grazing rate R, in their general formulation, i.e., for several prey (in biomass, mmolC m^-3^). All models include the same multiple prey size classes. Bold numbers in the first column indicate cases presented and discussed in the core paper, other cases are presented in supporting information. Λ is the rate at which saturation of the ingestion is achieved with increasing food level. *T* is the zooplankton feeding threshold. *a*_*T*_ and *b*_*T*_ represent allometric parameters for the size-dependant feeding threshold.
(DOCX)

S2 Table Biomasses (mmolC m^-3^). Biomasses are averaged over the 6 last years of the numerical experiments and spatially computed over the full depth, excluding up to 450 km (30 grid points) at the edge of the modelled domain to remove noisy signals generated by boundary forcing. If biomass is below a threshold of 10^−5^ mmol C m^-3^ on each cell for a given plankton type, we assume that the type does not emerge (no number is given in the table below). Bold cases represent cases showed in the core paper.
(DOCX)

S3 Table Grazing fluxes (mmolC m^-3^ d^-1^). Grazing fluxes are averaged over the 6 last years of the numerical experiments and spatially computed over the full depth, excluding up to 450 km (30 grid points) at the edge of the modelled domain to remove noisy signals generated by boundary forcing. If a plankton type (prey or predator) does not emerge (see S1 Table), there is not available flux. Bold cases represent cases showed in the core paper.
(DOCX)
