# Supplementary material for: On the sensitivity of plankton ecosystem models to the formulation of zooplankton grazing
Source: PLoS One. 2021 May 25;16(5):e0252033. doi: 10.1371/journal.pone.0252033 (PMC8148333; doi:10.1371/journal.pone.0252033)
Supplement: S1 Fig — Schematic representation of (a) ingestion rate and (b) clearance rate for a classic sigmoidal functional response (solid line) vs. a response considering a feeding threshold Ti and Tc, (for ingestion rate and for clearance rate, respectively) (dashed line). (DOCX) [file pone.0252033.s001.docx]

**
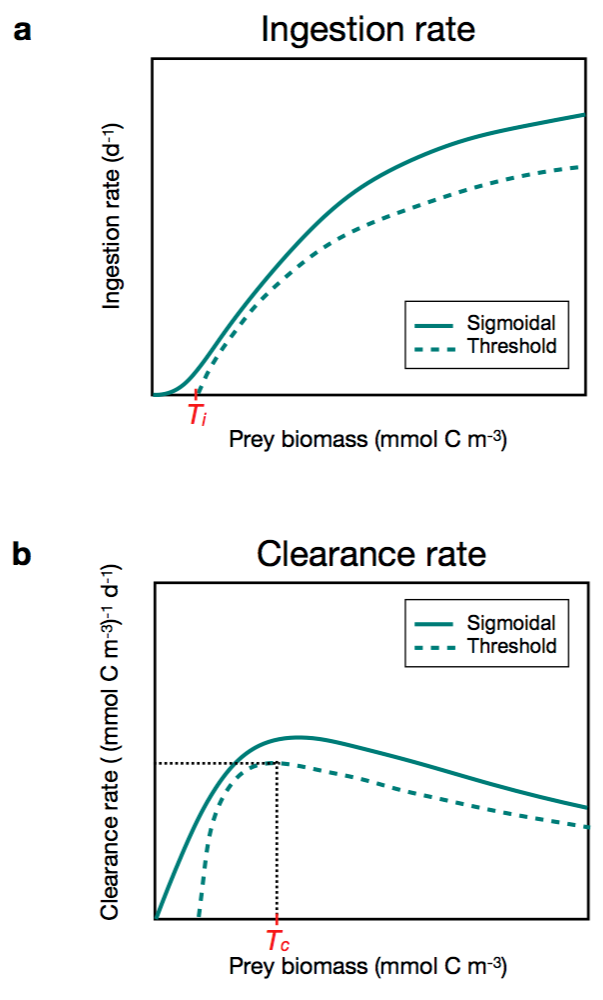
**

**S1 Fig**. Schematic representation of (a) ingestion rate and (b) clearance rate for a classic sigmoidal functional response (solid line) vs. a response considering a feeding threshold *T_i_* and *T_c_*, (for ingestion rate and for clearance rate, respectively) (dashed line).
