# Supplementary material for: On the sensitivity of plankton ecosystem models to the formulation of zooplankton grazing
Source: PLoS One. 2021 May 25;16(5):e0252033. doi: 10.1371/journal.pone.0252033 (PMC8148333; doi:10.1371/journal.pone.0252033)
Supplement: S2 Fig — (DOCX) [file pone.0252033.s002.docx]

**
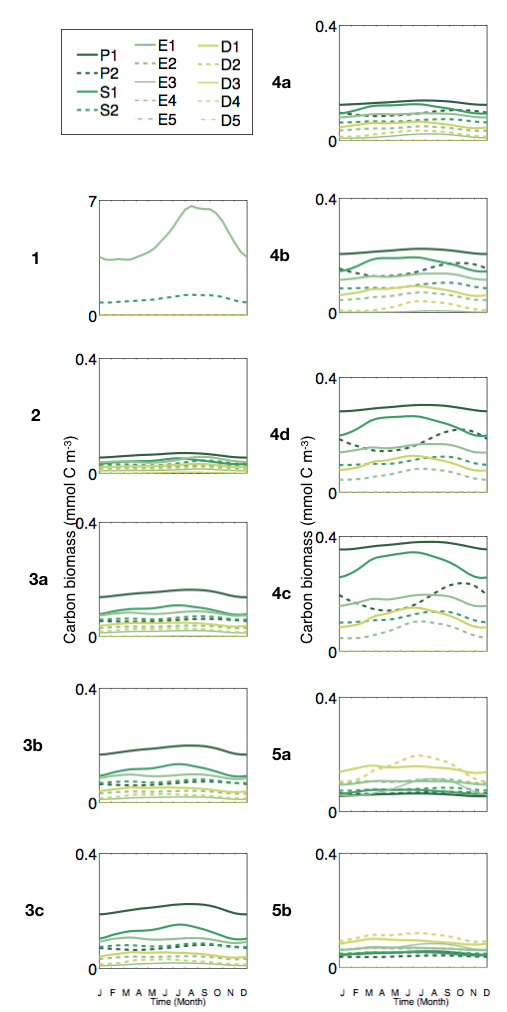
**

**S2 Fig.** Seasonal variation of detailed pfts accounting for emergent phytoplankton size classes (in  mmol  C  m^- 3^).
