# Supplementary material for: On the sensitivity of plankton ecosystem models to the formulation of zooplankton grazing
Source: PLoS One. 2021 May 25;16(5):e0252033. doi: 10.1371/journal.pone.0252033 (PMC8148333; doi:10.1371/journal.pone.0252033)
Supplement: S3 Fig — Seasonal variation of (column 1) the 4 plankton functional types (mmol C m-3) and (column 2) detailed size classes of zooplankton (mmol C m-3). (DOCX) [file pone.0252033.s003.docx]

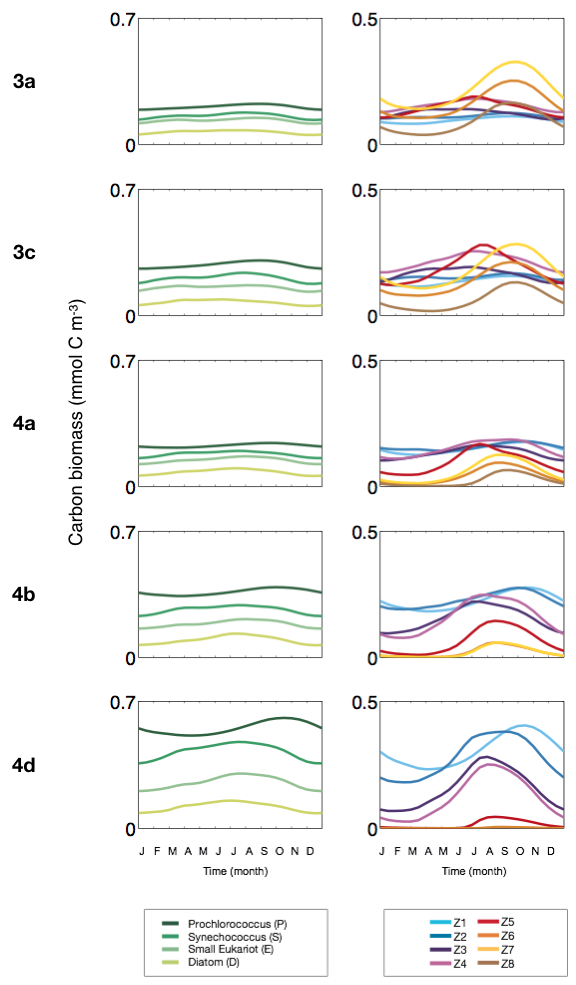


**S3 Fig.** Seasonal variation of (column 1) the 4 plankton functional types (mmol C m^-3^) and (column 2) detailed size classes of zooplankton (mmol C m^-3^).
