# Supplementary material for: On the sensitivity of plankton ecosystem models to the formulation of zooplankton grazing
Source: PLoS One. 2021 May 25;16(5):e0252033. doi: 10.1371/journal.pone.0252033 (PMC8148333; doi:10.1371/journal.pone.0252033)
Supplement: S4 Fig — Grazing fluxes correspond to equation 2. Color code corresponds to each zooplankton size class (as in Fig 3) while dotted-dashed, dashed and solid lines correspond to preys of increasing maximum growth and grazing rates, respectively (y-axis in mmol C m-3 d-1). (DOCX) [file pone.0252033.s004.docx]

**
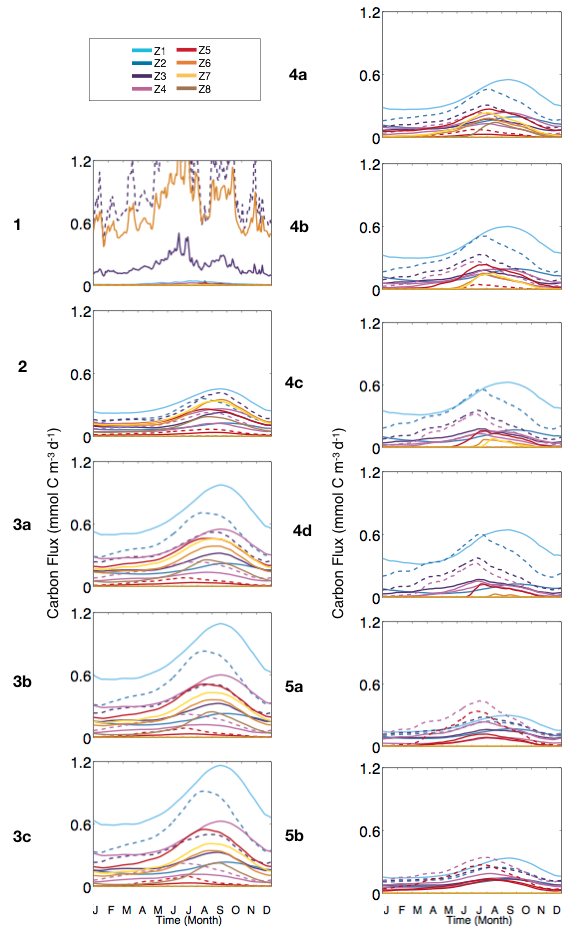
**

**S4 Fig.** Seasonal variation of grazing fluxes (averaged on the last year of simulation, integrated over depths and averaged over longitude and latitude, excluding boundary conditions). Grazing fluxes correspond to equation 2. Color code corresponds to each zooplankton size class (as in Fig. 3) while dotted-dashed, dashed and solid lines correspond to preys of increasing maximum growth and grazing rates, respectively (y- axis in  mmol C m^-3^ d^-1^).
