# Supplementary material for: On the sensitivity of plankton ecosystem models to the formulation of zooplankton grazing
Source: PLoS One. 2021 May 25;16(5):e0252033. doi: 10.1371/journal.pone.0252033 (PMC8148333; doi:10.1371/journal.pone.0252033)
Supplement: S5 Fig — (column 1) Annual mean surface chlorophyll-a concentration (mg Chl-a m-3); (column 2) Vertical sections of annual mean chlorophyll-a concentration across CalCOFI line 70 (mg Chl-a m-3). Results correspond to model outputs for grazing cases 3 and 4 not shown in the core paper. (DOCX) [file pone.0252033.s005.docx]

**
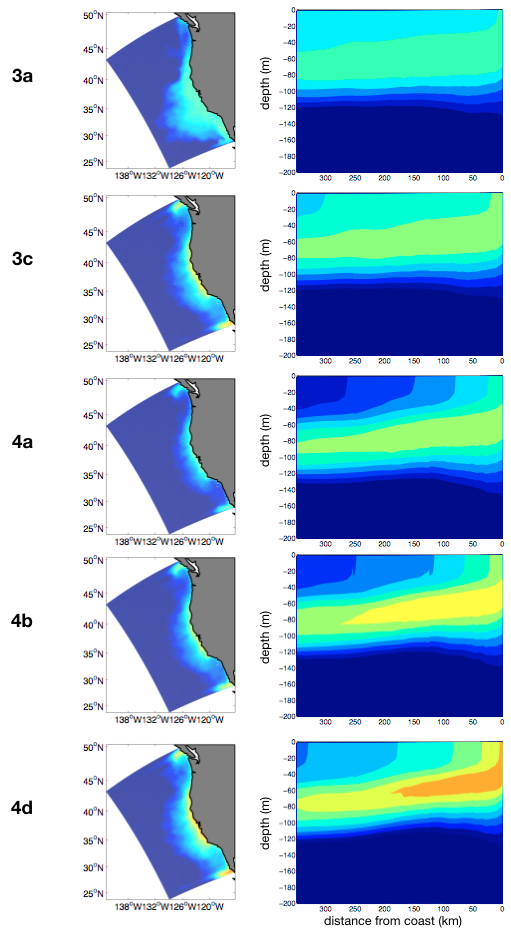
**

**S5 Fig.** (column 1) Annual mean surface chlorophyll-a concentration (mg Chl-a m^-3^); (column 2) Vertical sections of annual mean chlorophyll-a concentration across CalCOFI line 70 (mg Chl-a m-3). Results correspond to model outputs for grazing cases 3 and 4 not shown in the core paper.
