# Supplementary material for: On the sensitivity of plankton ecosystem models to the formulation of zooplankton grazing
Source: PLoS One. 2021 May 25;16(5):e0252033. doi: 10.1371/journal.pone.0252033 (PMC8148333; doi:10.1371/journal.pone.0252033)
Supplement: S6 Fig — (column 1) Seasonal variation of total phytoplankton biomass (blue), total zooplankton biomass (red) and the associated Z:P ratio (black). The number in the upper-left corner indicates the seasonal mean of the Z:P ratio. (column 2) Spatial distribution of the annual-mean Z:P ratio. The solid black line indicates Z:P = 1. Results correspond to model outputs for grazing cases 3 and 4 not shown in the core paper. (DOCX) [file pone.0252033.s006.docx]

**
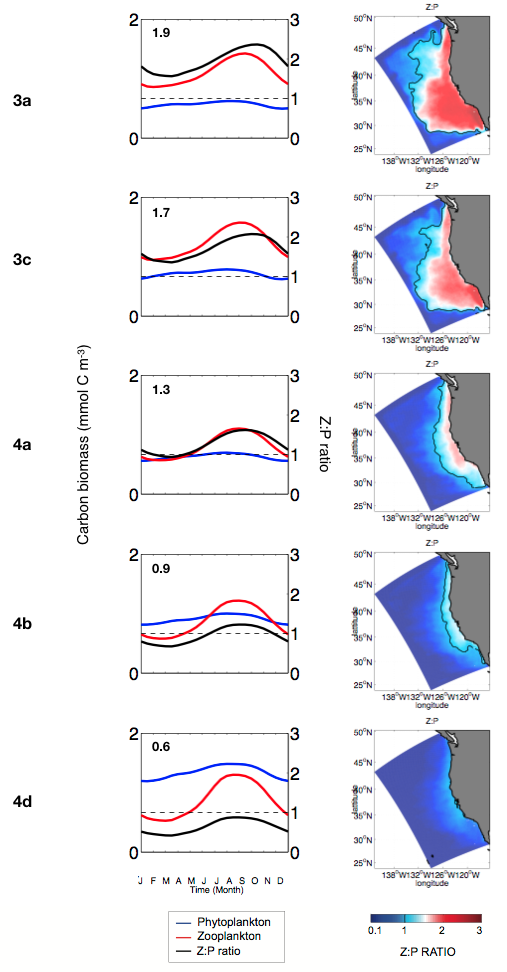
**

**S6 Fig.** Z:P ratio. (column 1) Seasonal variation of total phytoplankton biomass (blue), total zooplankton biomass (red) and the associated Z:P ratio (black). The number in the upper-left corner indicates the seasonal mean of the Z:P ratio. (column 2) Spatial distribution of the annual-mean Z:P ratio. The solid black line indicates Z:P =1. Results correspond to model outputs for grazing cases 3 and 4 not shown in the core paper.
