# Supplementary material for: On the sensitivity of plankton ecosystem models to the formulation of zooplankton grazing
Source: PLoS One. 2021 May 25;16(5):e0252033. doi: 10.1371/journal.pone.0252033 (PMC8148333; doi:10.1371/journal.pone.0252033)
Supplement: S7 Fig — (columns 1 and 2) Richness and (column 3 and 4) niches of phytoplankton (columns 1 and 3) and zooplankton (columns 2 and 4) for grazing in cases 3 and 4. (DOCX) [file pone.0252033.s007.docx]

**
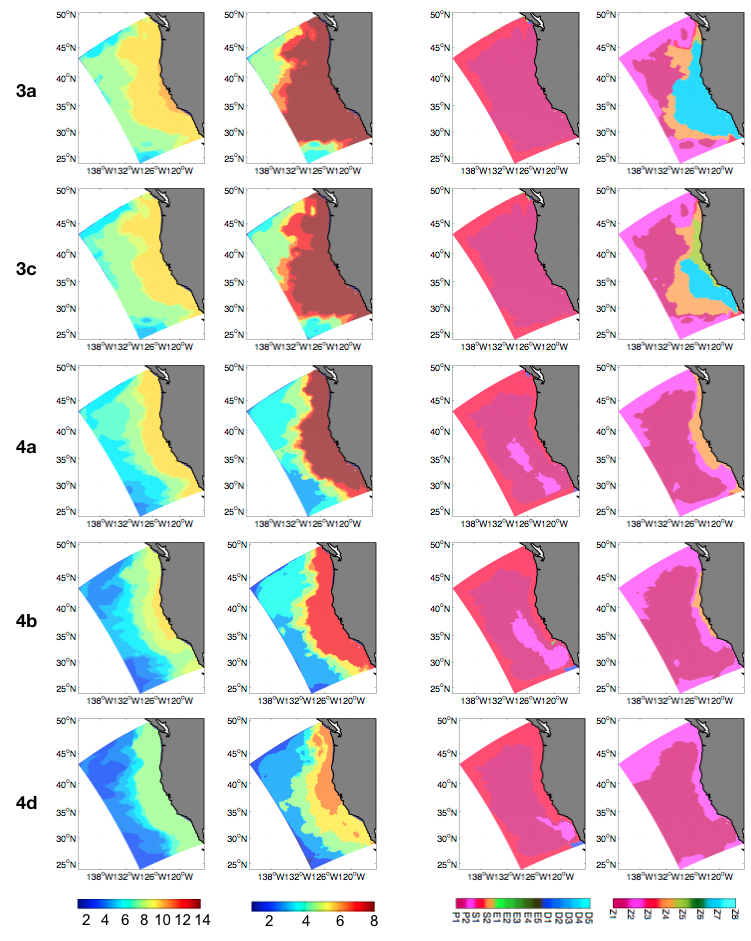
S7 Fig.** (columns 1 and 2) Richness and (column 3 and 4) niches of phytoplankton (columns 1 and 3) and zooplankton (columns 2 and 4) for grazing in cases 3 and 4.
