# Supplementary material for: On the sensitivity of plankton ecosystem models to the formulation of zooplankton grazing
Source: PLoS One. 2021 May 25;16(5):e0252033. doi: 10.1371/journal.pone.0252033 (PMC8148333; doi:10.1371/journal.pone.0252033)
Supplement: S8 Fig — (DOCX) [file pone.0252033.s008.docx]

**
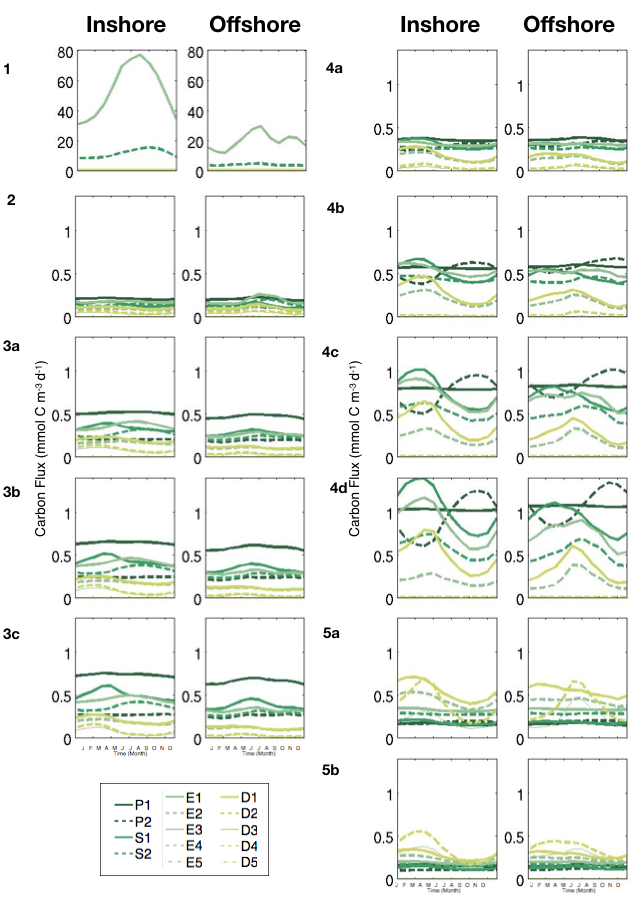
**

**S8 Fig.** Comparison of phytoplankton concentrations between coastal (0-100km) and offshore region (300- 400km), in the Southern CCS (+/- 0.5˚ around line 90 of CalCOFI): seasonal variation of detailed pfts accounting for emergent phytoplankton size classes (in mmol C m^-3^).
