# Supplementary material for: On the sensitivity of plankton ecosystem models to the formulation of zooplankton grazing
Source: PLoS One. 2021 May 25;16(5):e0252033. doi: 10.1371/journal.pone.0252033 (PMC8148333; doi:10.1371/journal.pone.0252033)
Supplement: S10 Fig — The number in the upper-left corner indicat. (DOCX) [file pone.0252033.s010.docx]

**
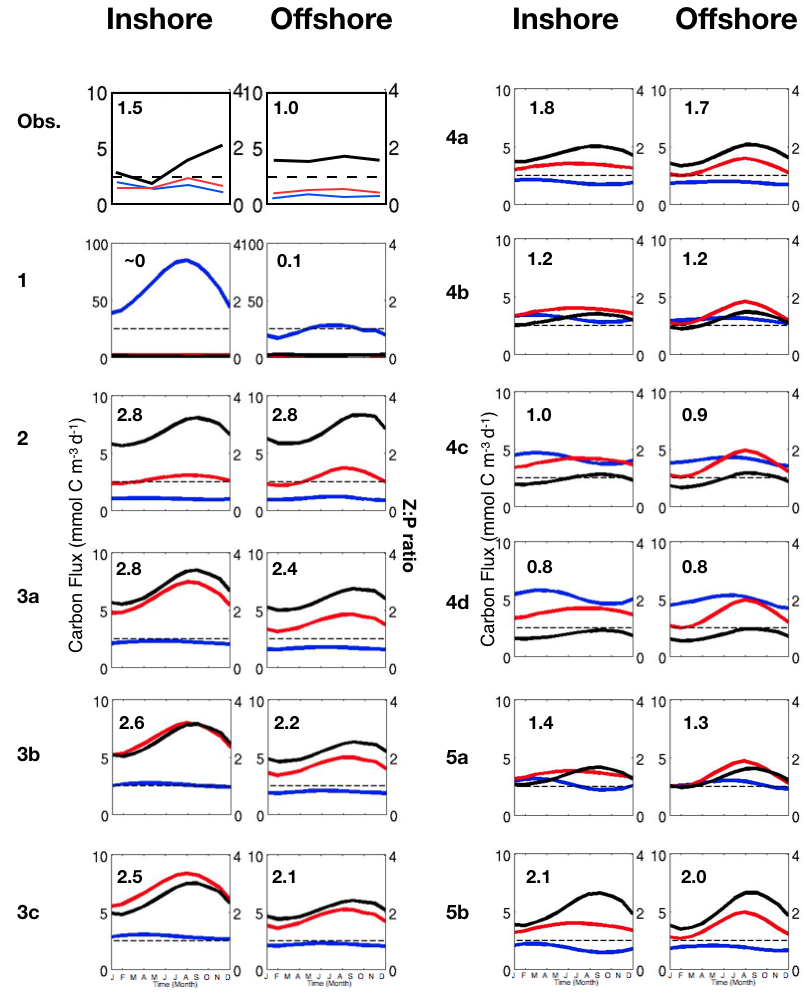
**

**S10 Fig.** Comparison of Z:P ratio between coastal (0-100km) and offshore region (300-400km), in the Southern CCS (+/- 0.5˚ around line 90 of CalCOFI): Seasonal variation of total phytoplankton biomass (blue), total zooplankton biomass (red) and the associated Z:P ratio (black) from observations (CalCOFI data) and the different numerical experiments. The number in the upper-left corner indicates the seasonal mean of the Z:P ratio.
