# Supplementary material for: On the sensitivity of plankton ecosystem models to the formulation of zooplankton grazing
Source: PLoS One. 2021 May 25;16(5):e0252033. doi: 10.1371/journal.pone.0252033 (PMC8148333; doi:10.1371/journal.pone.0252033)
Supplement: S1 Table — All models include the same multiple prey size classes. Bold numbers in the first column indicate cases presented and discussed in the core paper, other cases are presented in supporting information. Λ is the rate at which saturation of the ingestion is achieved with increasing food level. T is the zooplankton feeding threshold. aT and bT represent allometric parameters for the size-dependant feeding threshold. (DOCX) [file pone.0252033.s011.docx]

**S1 Table.** Various mathematical formulations of the grazing rate R, in their general formulation, i.e., for several prey (in biomass, mmol C m^-3^). All models include the same multiple prey size classes. Bold numbers in the first column indicate cases presented and discussed in the core paper, other cases are presented in supporting information. $\Lambda$ is the rate at which saturation of the ingestion is achieved with increasing food level. $T$ is the zooplankton feeding threshold. $a_{T}$ and $b_{T}$ represent allometric parameters for the size-dependant feeding threshold.

| **Case** | **Grazing refuge**  **function** | **R mathematical formulation in equation (2)** |
| --- | --- | --- |
| **1** | No refuge | $R\left( X_{jprey} \right)=\frac{{\phi_{jpred,jprey}X}_{jprey}}{\mathcal{F}_{jpred}+k_{jpred}}$  $\mathcal{F}_{jpred}=\sum_{jprey=1}^{J} {\phi_{jpred,jprey}X}_{jprey}$  and $\phi_{jpred,jprey}$ the prey palatability |
| **2** | Ivlev (Iv) | $R(X_{jprey})=\frac{{\phi_{jpred,jprey}X}_{jprey}}{\mathcal{F}_{jpred}+k_{jpred}}\left( 1-e^{\Lambda\mathcal{F}_{jpred}} \right)$ |
| 3a | Sigmoid (Sig) | $R(X_{jprey})=\frac{{\phi_{jpred,jprey}X}_{jprey}}{\mathcal{F}_{jpred}+k_{jpred}}\frac{{({\phi_{jpred,jprey}X}_{jprey})}^{2}}{{\mathcal{F}_{jpred}}^{2}+K^{2}}$ |
| **3b** |  |  |
| 3c |  |  |
| 4a | Ivlev Size-Independent-Threshold (Iv-SIT) | $R(X_{jprey})=\left\{ \begin{aligned} \frac{{\phi_{jpred,jprey}X}_{jprey}-T}{\left( \mathcal{F}_{jpred}-T \right)+k_{jpred}}\left( 1-e^{\Lambda\left( \mathcal{F}_{jpred}-T \right)} \right), X>T \\ 0, X\leq T \end{aligned} \right.$ |
| 4b |  |  |
| **4c** |  |  |
| 4d |  |  |
| **5a** | Ivlev Size-Dependent-Threshold (Iv-SDT) | $R(X_{jprey})=\left\{ \begin{aligned} \frac{{\phi_{jpred,jprey}X}_{jprey}-T}{\left( \mathcal{F}_{jpred}-T \right)+k_{jpred}}\left( 1-e^{\Lambda\left( \mathcal{F}_{jpred}-T \right)} \right), X>T \\ 0, X\leq T \end{aligned} \right.$  with $T=a_{T}V^{b_{T}}$ |
| **5b** |  |  |
