# Supplementary material for: On the sensitivity of plankton ecosystem models to the formulation of zooplankton grazing
Source: PLoS One. 2021 May 25;16(5):e0252033. doi: 10.1371/journal.pone.0252033 (PMC8148333; doi:10.1371/journal.pone.0252033)
Supplement: S2 Table — Biomasses are averaged over the 6 last years of the numerical experiments and spatially computed over the full depth, excluding up to 450 km (30 grid points) at the edge of the modelled domain to remove noisy signals generated by boundary forcing. If biomass is below a threshold of 10−5 mmol C m-3 on each cell for a given plankton type, we assume that the type does not emerge (no number is given in the table below). Bold cases represent cases showed in the core paper. (DOCX) [file pone.0252033.s012.docx]

**S2 Table**. Biomasses (mmolC m^-3^). Biomasses are averaged over the 6 last years of the numerical experiments and spatially computed over the full depth, excluding up to 450 km (30 grid points) at the edge of the modelled domain to remove noisy signals generated by boundary forcing. If biomass is below a threshold of 10^-5^ mmol C m^-3^ on each cell for a given plankton type, we assume that the type does not emerge (no number is given in the table below). Bold cases represent cases showed in the core paper.

| Cases  Biomasses | **1** | **2** | 3a | **3b** | 3c | 4a | 4b | **4c** | 4d | **5a** | **5b** |
| --- | --- | --- | --- | --- | --- | --- | --- | --- | --- | --- | --- |
| P1 | 6,3e-03 | 6,4e-02 | 1,5e-01 | 1,9e-01 | 2,1e-01 | 1,3e-01 | 2,1e-01 | 2,9e-01 | 3,7e-01 | 5,9e-02 | 5,1e-02 |
| P2 | 1,8e-03 | 2,6e-02 | 5,7e-02 | 6,7e-02 | 7,3e-02 | 9,5e-02 | 1,5e-01 | 1,8e-01 | 1,8e-01 | 6,5e-02 | 3,9e-02 |
| S1 | 3,1e-03 | 4,2e-02 | 9,4e-02 | 1,1e-01 | 1,3e-01 | 1,1e-01 | 1,7e-01 | 2,4e-01 | 3,1e-01 | 7,1e-02 | 5,0e-02 |
| S2 | 1,0e+00 | 3,5e-02 | 6,4e-02 | 7,4e-02 | 8,0e-02 | 6,8e-02 | 9,2e-02 | 1,1e-01 | 1,2e-01 | 7,8e-02 | 5,2e-02 |
| E1 | 4,8e+00 | 4,5e-02 | 8,1e-02 | 9,3e-02 | 1,0e-01 | 8,9e-02 | 1,3e-01 | 1,6e-01 | 1,8e-01 | 1,0e-01 | 6,7e-02 |
| E2 | 5,3e-04 | 2,1e-02 | 3,5e-02 | 3,7e-02 | 3,9e-02 | 4,2e-02 | 5,7e-02 | 6,2e-02 | 7,3e-02 | 1,1e-01 | 6,6e-02 |
| E3 | 9,9e-05 | 1,2e-02 | 1,7e-02 | 1,6e-02 | 1,5e-02 | 1,5e-02 | 3,0e-03 | 4,5e-06 | 1,3e-07 | 8,3e-02 | 7,2e-02 |
| E4 | - | 5,9e-04 | 1,5e-06 | - | - | - | - | - | - | - | - |
| E5 | - | - | - | - | - | - | - | - | - | - | - |
| D1 | 1,4e-03 | 2,7e-02 | 4,4e-02 | 4,7e-02 | 5,0e-02 | 5,6e-02 | 7,7e-02 | 1,0e-01 | 1,2e-01 | 1,5e-01 | 9,1e-02 |
| D2 | 5,5e-04 | 1,6e-02 | 2,4e-02 | 2,3e-02 | 2,4e-02 | 2,4e-02 | 2,1e-02 | 8,2e-04 | 9,7e-06 | 1,5e-01 | 1,1e-01 |
| D3 | - | 2,2e-03 | 1,1e-03 | 2,7e-06 | - | - | - | - | - | - | 5,8e-05 |
| D4 | - | - | - | - | - | - | - | - | - | - | - |
| D5 | - | - | - | - | - | - | - | - | - | - | - |
| Z1 | 3,8e-03 | 4,5e-02 | 9,6e-02 | 1,2e-01 | 1,3e-01 | 1,5e-01 | 2,2e-01 | 2,7e-01 | 3,1e-01 | 1,2e-01 | 8,7e-02 |
| Z2 | 3,4e-03 | 4,9e-02 | 1,1e-01 | 1,4e-01 | 1,5e-01 | 1,6e-01 | 2,3e-01 | 2,7e-01 | 2,8e-01 | 1,9e-01 | 1,4e-01 |
| Z3 | 1,7e-02 | 5,1e-02 | 1,2e-01 | 1,5e-01 | 1,6e-01 | 1,3e-01 | 1,5e-01 | 1,6e-01 | 1,6e-01 | 2,1e-01 | 2,1e-01 |
| Z4 | 8,0e-03 | 7,1e-02 | 1,5e-01 | 1,9e-01 | 2,1e-01 | 1,5e-01 | 1,6e-01 | 1,5e-01 | 1,2e-01 | 2,8e-01 | 3,1e-01 |
| Z5 | 5,5e-03 | 7,0e-02 | 1,4e-01 | 1,7e-01 | 1,8e-01 | 9,5e-02 | 6,3e-02 | 2,5e-02 | 1,4e-02 | 1,3e-01 | 1,9e-01 |
| Z6 | 5,9e-01 | 1,4e-01 | 1,6e-01 | 1,4e-01 | 1,3e-01 | 4,2e-02 | 2,0e-02 | 6,8e-03 | 1,3e-03 | - | - |
| Z7 | 3,8e-03 | 1,5e-01 | 2,2e-01 | 2,0e-01 | 1,8e-01 | 5,7e-02 | 2,0e-02 | 5,0e-03 | - | - | - |
| Z8 | 1,5e-03 | 7,2e-02 | 9,0e-02 | 7,3e-02 | 6,1e-02 | 2,2e-02 | - | - | - | - | - |
