# Supplementary material for: On the sensitivity of plankton ecosystem models to the formulation of zooplankton grazing
Source: PLoS One. 2021 May 25;16(5):e0252033. doi: 10.1371/journal.pone.0252033 (PMC8148333; doi:10.1371/journal.pone.0252033)
Supplement: S3 Table — Grazing fluxes are averaged over the 6 last years of the numerical experiments and spatially computed over the full depth, excluding up to 450 km (30 grid points) at the edge of the modelled domain to remove noisy signals generated by boundary forcing. If a plankton type (prey or predator) does not emerge (see S1 Table), there is not available flux. Bold cases represent cases showed in the core paper. (DOCX) [file pone.0252033.s013.docx]

**S3 Table**. Grazing Fluxes (mmolC m^-3^ d^-1^). Grazing fluxes are averaged over the 6 last years of the numerical experiments and spatially computed over the full depth, excluding up to 450 km (30 grid points) at the edge of the modelled domain to remove noisy signals generated by boundary forcing. If a plankton type (prey or predator) does not emerge (see Table S1), there is not available flux. Bold cases represent cases showed in the core paper.

| Cases Flux Predator \| Prey | | **1** | | **2** | | 3a | | **3b** | | 3c | | 4a | | 4b | | **4c** | | 4d | | **5a** | | **5b** | |  |
| --- | --- | --- | --- | --- | --- | --- | --- | --- | --- | --- | --- | --- | --- | --- | --- | --- | --- | --- | --- | --- | --- | --- | --- | --- |
| Z1 | P1 | | 2,0e-2 | | 3,1e-1 | | 6,9e-1 | | 7,7e-1 | | 8,2e-1 | | 3,8e-1 | | 4,2e-1 | | 4,5e-1 | | 4,6e-1 | | 2,1e-1 | | 2,2e-1 | |
| Z2 | P2 | | 3,1e-3 | | 8,7e-2 | | 1,6e-1 | | 1,7e-1 | | 1,7e-1 | | 1,3e-1 | | 1,3e-1 | | 1,0e-1 | | 7,7e-2 | | 1,2e-1 | | 9,4e-2 | |
| Z2 | S1 | | 8,4e-3 | | 2,2e-1 | | 4,7e-1 | | 5,3e-1 | | 5,7e-1 | | 2,8e-1 | | 3,1e-1 | | 3,4e-1 | | 3,6e-1 | | 1,6e-1 | | 1,7e-1 | |
| Z3 | S2 | | 1,9e-1 | | 1,3e-1 | | 2,1e-1 | | 2,2e-1 | | 2,2e-1 | | 1,1e-1 | | 1,1e-1 | | 1,0e-1 | | 9,2e-2 | | 1,1e-1 | | 1,0e-1 | |
| Z3 | E1 | | 1,0e+0 | | 2,4e-1 | | 3,5e-1 | | 3,5e-1 | | 3,5e-1 | | 1,8e-1 | | 1,8e-1 | | 1,9e-1 | | 1,9e-1 | | 1,8e-1 | | 1,7e-1 | |
| Z4 | E2 | | 4,2e-4 | | 7,3e-2 | | 9,2e-2 | | 8,4e-2 | | 8,0e-2 | | 6,9e-2 | | 6,8e-2 | | 5,7e-2 | | 5,7e-2 | | 1,4e-1 | | 1,2e-1 | |
| Z4 | D1 | | 3,0e-3 | | 1,3e-1 | | 1,6e-1 | | 1,4e-1 | | 1,4e-1 | | 1,2e-1 | | 1,2e-1 | | 1,4e-1 | | 1,3e-1 | | 2,5e-1 | | 2,1e-1 | |
| Z4 | Z1 | | 1,1e-2 | | 1,8e-1 | | 3,8e-1 | | 4,1e-1 | | 4,3e-1 | | 1,5e-1 | | 1,1e-1 | | 8,3e-2 | | 6,0e-2 | | 5,0e-2 | | 9,0e-2 | |
| Z5 | E3 | | 2,9e-5 | | 2,1e-2 | | 2,3e-2 | | 1,8e-2 | | 1,6e-2 | | 1,2e-2 | | 2,6e-4 | | 2,1E-17 | | 1,7E-17 | | 4,9e-2 | | 7,2e-2 | |
| Z5 | D2 | | 6,4e-4 | | 3,9e-2 | | 4,4e-2 | | 3,9e-2 | | 3,8e-2 | | 2,9e-2 | | 1,2e-2 | | 1,9e-8 | | 9,4E-17 | | 1,4e-1 | | 1,4e-1 | |
| Z5 | Z2 | | 5,1e-3 | | 1,7e-1 | | 3,1e-1 | | 3,2e-1 | | 3,3e-1 | | 1,4e-1 | | 9,3e-2 | | 4,7e-2 | | 3,0e-2 | | 3,6e-2 | | 6,9e-2 | |
| Z6 | E4 | | - | | 1,6e-4 | | - | | - | | - | | - | | - | | - | | - | | - | | - | |
| Z6 | D3 | | - | | 1,8e-3 | | 2,2e-4 | | - | | - | | - | | - | | - | | - | | - | | - | |
| Z6 | Z3 | | 7,4e-1 | | 1,9e-1 | | 2,3e-1 | | 2,1e-1 | | 2,0e-1 | | 7,1e-2 | | 4,1e-2 | | 1,7e-2 | | 4,3e-3 | | - | | - | |
| Z7 | E5 | | - | | - | | - | | - | | - | | - | | - | | - | | - | | - | | - | |
| Z7 | D4 | | - | | - | | - | | - | | - | | - | | - | | - | | - | | - | | - | |
| Z7 | Z4 | | 3,7e-3 | | 2,0e-1 | | 2,9e-1 | | 2,6e-1 | | 2,5e-1 | | 9,3e-2 | | 4,0e-2 | | 1,2e-2 | | - | | - | | - | |
| Z8 | D5 | | - | | - | | - | | - | | - | | - | | - | | - | | - | | - | | - | |
| Z8 | Z5 | | 1,1e-3 | | 9,5e-2 | | 1,2e-1 | | 1,0e-1 | | 8,7e-2 | | 3,6e-2 | | - | | - | | - | | - | | - | |
